# The potential of dietary treatment in patients with glycogen storage disease type IV

**DOI:** 10.1002/jimd.12339

**Published:** 2020-12-21

**Authors:** Terry G. J. Derks, Fabian Peeks, Foekje de Boer, Marieke Fokkert‐Wilts, Hubert P. J. van der Doef, Marius C. van den Heuvel, Edyta Szymańska, Dariusz Rokicki, Patrick T. Ryan, David A. Weinstein

**Affiliations:** ^1^ Department of Metabolic Diseases Beatrix Children's Hospital, University Medical Centre Groningen, University of Groningen Groningen the Netherlands; ^2^ Department of Pediatric Gastroenterology Hepatology and Nutrition Beatrix Children's Hospital, University Medical Centre Groningen, University of Groningen Groningen the Netherlands; ^3^ Department of Pathology & Medical Biology, Pathology Section, University of Groningen University Medical Center Groningen Hanzeplein Groningen Netherlands; ^4^ Department of Gastroenterology, Hepatology, Feeding Disorders and Pediatrics The Childrens' Memorial Health Institute Warsaw Poland; ^5^ Department of Pediatrics, Nutrition and Metabolic Disorders The Childrens' Memorial Health Institute Warsaw Poland; ^6^ Glycogen Storage Disease Program, Connecticut Children's Medical Center Hartford Connecticut USA; ^7^ Department of Pediatrics University of Connecticut Health Center Farmington Connecticut USA

**Keywords:** dietary intervention, glycogen storage disease, glycogen storage disease type IV, inherited metabolic disease, liver transplantation

## Abstract

There is paucity of literature on dietary treatment in glycogen storage disease (GSD) type IV and formal guidelines are not available. Traditionally, liver transplantation was considered the only treatment option for GSD IV. In light of the success of dietary treatment for the other hepatic forms of GSD, we have initiated this observational study to assess the outcomes of medical diets, which limit the accumulation of glycogen. Clinical, dietary, laboratory, and imaging data for 15 GSD IV patients from three centres are presented. Medical diets may have the potential to delay or prevent liver transplantation, improve growth and normalize serum aminotransferases. Individual care plans aim to avoid both hyperglycaemia, hypoglycaemia and/or hyperketosis, to minimize glycogen accumulation and catabolism, respectively. Multidisciplinary monitoring includes balancing between traditional markers of metabolic control (ie, growth, liver size, serum aminotransferases, glucose homeostasis, lactate, and ketones), liver function (ie, synthesis, bile flow and detoxification of protein), and symptoms and signs of portal hypertension.

## Graphical abstract

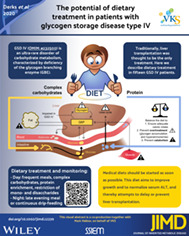

**Abbreviations**
ACTN2: actinin alpha 2
AP: alkaline phosphatase
APTT: activated partial thromboplastin time
ALT: alanine aminotransferase
AST: aspartate aminotransferase
CGM: continuous glucose monitoring
CK: creatinine kinase
CK‐MB: creatine kinase isoenzyme muscle‐brain
CMHI: The Children's Memorial Health Institute
CMV: cytomegalovirus
CNGDF: continuous nocturnal gastric drip feeding
ECG: electrocardiogram
F: female
Fam: family
FI: fasting intolerance
FTT: failure to thrive
GBE: glycogen branching enzyme
GGT: gamma‐glutamyl transferase
GSD: glycogen storage disease
GS‐MS: gas chromatography‐mass spectrometry
HBV: hepatitis B virus
HCV: hepatitis C virus
HELLP: hemolysis, elevated liver enzymes, low platelet count
HIV: human immunodeficiency virus
HK: hyperketosis
HM: hepatomegaly
HSM: hepatosplenomegaly
H&E: hematoxylin and eosin stain
INR: International Normalized Ratio
IRB: Independent Review Board
LEM: late evening meal
LT: liver transplantation
M: male
METc: Medical Ethical Committee
NGS: next generation sequencing
nm: not measured
np: not performed
NT‐proBNP: *N*‐terminal pro hormone brain natriuretic peptide
OMIM: Online Mendelian Inheritance in Man
P: patient
PAS: periodic acid‐Schiff
PAS‐D: periodic acid‐Schiff after digestion
PE: protein enrichment
PEG: percutaneous endoscopic gastrostomy
PHKB: Phosphorylase Kinase Regulatory Subunit Beta
PT: prothrombin time
mRNA: messenger ribonucleic acid
SD: standard deviation
SMA: spinal muscular atrophy
UCCS: uncooked cornstarch
UMCG: University Medical Centre Groningen
US: ultra sound

## 1 INTRODUCTION

Glycogen storage disease (GSD) type IV (GSD IV, OMIM #232500) is a rare inherited disorder of carbohydrate metabolism first described by Andersen in 1956 as “familial cirrhosis of the liver with storage of abnormal glycogen”.^1^ The disease is caused by autosomal recessive mutations in the *GBE1* gene (OMIM *607839), which leads to 1,4‐α‐glucan‐branching enzyme (ie, glycogen branching enzyme, GBE) deficiency. GBE deficiency causes the production of relatively insoluble glycogen of abnormal structure with fewer branch points, more α‐1‐4‐linked glucose units, and longer outer chains than normal glycogen. The prevalence of GSD IV is estimated 1 in 600  000 to 800 000, but this was before next generation sequencing (NGS) became available.^2^

Clinical presentation of GSD IV patients is extremely heterogeneous and involves the liver, the neuromuscular system and the heart.^2^, ^3^, ^4^, ^5^ In the classical (progressive) hepatic subtype, children are normal at birth, but develop hepatomegaly, hypotonia, and developmental delay during their first months. The disease then rapidly progresses to liver cirrhosis with portal hypertension and ascites between the second and fourth years of life, ultimately causing death in early childhood.^1^ Currently, liver transplantation (LT) is considered the only treatment for patients with the progressive hepatic subtype of GSD IV.^6^, ^7^ A nonprogressing hepatic form has been reported in a few cases.^8^, ^9^, ^10^ Neuromuscular presentations' onset may range from fetal to adult age. The most severe form starts before birth with decrease or absence of fetal movements, arthrogryposis, hypoplastic lungs, and may cause perinatal death. Adult polyglucosan body disease results in the accumulation of polyglucosan bodies in muscle, nerve, and various other tissues of the body.^11^ Hence, it may be wiser to consider GSD IV as a phenotypic continuum, with different degrees of involvement of each organ system, rather than splitting the disease in subtypes.^12^

Classical symptoms and signs of patients with hepatic GSD include fasting intolerance, failure to thrive and hepatomegaly, biochemically characterized by fasting hypoglycaemia, increased serum aminotransferases, and hyperlipidaemia.^13^ Dietary treatment is the cornerstone of management aiming at maintenance of euglycaemia, prevention of secondary metabolic perturbations, and long‐term complications affecting multiple organs, such as the liver (hepatocellular adenomas and carcinomas), kidneys (proteinuria, renal insufficiency, stones), heart (cardiomyopathy), muscle (myopathy), and bone (osteopenia, osteoporosis). Dietary treatment for hepatic GSD may include GSD subtype‐specific and age‐dependent combinations of frequent meals, a late evening meal (LEM), continuous nocturnal gastric drip feeding (CNGDF), restriction of mono‐ and disaccharides, addition of uncooked cornstarch (UCCS), and protein enrichment (PE).^14^

There is a paucity of literature for dietary treatment in GSD IV. Most case reports lack detailed information on the medical diets and formal guidelines are not available. We previously employed the strategy of priority setting partnership for stakeholder participation and patient empowerment of hepatic GSD.^15^ For the GSD IV stakeholders, the top three research priorities refer to (a) natural history, (b) indications for liver transplantation, and (c) dietary restrictions. Therefore, we report a multicentre, retrospective, observational, longitudinal case series of clinical and laboratory data in 15 GSD IV patients with liver and neuromuscular phenotypes, demonstrating the potential of dietary treatment in these patients.

## 2 METHODS

### 2.1 Patients

The Medical Ethical Committee of the University Medical Center Groningen stated that the Medical Research Involving Human Subjects Act was not applicable and that official study approval by the Medical Ethical Committee was not required (METc 2019/119). The study was approved for waived consent as it concerned retrospective, anonymous data. In the United States, the data were collected as part of a natural history protocol with oversight from the Connecticut Children's IRB with signed consent from the parents (IRB# 17‐003). For the Polish patients, the data were collected as part of a natural history protocol and according to this no IRB's consent is required. One of the final versions of the manuscript was shared with the patients and/or parents for feedback and approval for submission.

Data were studied from all GSD IV patients followed by three centres: (a) the Section of Metabolic Diseases, Beatrix Children's Hospital, University Medical Centre Groningen (UMCG) in the Netherlands, (b) the Glycogen Storage Disease Program at Connecticut Children's in the United States, and (c) the Children's Memorial Health Institute (CMHI) in Warsaw, Poland.

Patients were selected based on either confirmatory enzymatic and/or *GBE1* genotypes/mutations, which are displayed according to the reference sequence NM_000158.4. Clinical case descriptions of P7^16^ and P12‐14^17^ were published previously.

### 2.2 Clinical and biochemical data

This was a multicentre, retrospective, observational, longitudinal case series of GSD IV patients. Longitudinal clinical, dietary, laboratory and imaging data were retrieved retrospectively from the paper and electronic source files before June 1, 2020.

Clinical parameters included biometry (height‐for‐age, weight‐for‐age, weight‐for height), liver and spleen size (cm below costal margin in the midclavicular line) in relation to the prescribed medical diet or diet history. For patients 1 to 11, biometrical data were compared with the Dutch TNO 2010 standard growth diagrams and analyzed with Growth Analyzer VE version 1.6.5.4. For patients 12 to 15, biometrical data were compared with the WHO standard growth diagrams. The diets were individually prescribed based on the age, weight, and laboratory parameters, such as preprandial capillary blood glucose and ketone concentrations, and parameters of liver damage and function.

Dietary parameters included type of dietary treatment, total energy, total protein (dietary protein, protein from supplements), total fat, total carbohydrates (including complex carbohydrates).

Laboratory parameters were compared to local reference values and included parameters of metabolic control (ie, glucose, lactate, uric acid, triglycerides, total cholesterol, 3‐hydroxybutyrate, acetoacetate, and serum aminotransferases), liver function studies including activated partial thromboplastin time (APTT), prothrombin time (PT), albumin, ammonia, total bilirubin, direct bilirubin, gamma‐glutamyl transferase (GGT), and alkaline phosphatase (AP), neuromuscular parameters including creatine kinase (CK) and cardiac parameters including *N*‐terminal pro hormone brain natriuretic peptide (NT‐proBNP). The definition of portal hypertension is adapted from clinically evident portal hypertension (CEPH) as either (a) thrombocytopenia (<150*10̂9/L) and splenomegaly (as diagnosed on US), or (b) one or more clinical manifestations of portal hypertension (such as ascites, endoscopic evidence of esophageal or gastric varices).^18^ Liver dysfunction is defined by abnormalities in liver function parameters including synthesis (APTT, PT, albumin), bile flow (total and direct bilirubin, GGT, and AP) and detoxification of protein (ammonia).

Imaging and function parameters included ECG, abdominal, and cardiac imaging (ultrasound, computed tomography, magnetic resonance imaging).

### 2.3 Histology

Paraffin‐embedded slides of diagnostic liver biopsies and liver explants were re‐evaluated. Slides were stained with hematoxylin and eosin (H&E), Masson trichrome, Periodic acid‐Schiff (PAS), and PAS after digestion (PAS‐D). The amount and distribution of fibrosis were scored with the Venturi scoring system, which discerns portal fibrosis, sinusoidal fibrosis and perivenular fibrosis.^19^ The Ishak scoring system for inflammation was used to evaluate the amount and distribution of inflammation.^20^ We evaluated the presence and the amount of eosinophilic cytoplasmic inclusions in hepatocytes with the PAS staining. The PAS‐D staining was added to identify GSD IV with atypical histological features.^21^

### 2.4 Statistics

Descriptive statistical analysis was performed using Microsoft Excel for Mac Version 15.19.1 and IBM SPSS Statistics 23. After testing for normality with the Kolmogorov‐Smirnov test, data between patients with and without liver transplantation were tested with the Mann‐Whitney *U* test. Data before and after dietary treatment were tested with the Wilcoxon Signed Ranks Test.

## 3 RESULTS

Table 1 summarized general characteristics of all 15 GSD IV patients, including the family of the patient, current age, if performed *GBE1* mutations and age at LT, signs and symptoms of the clinical phenotype, and a summary of the different prescribed medical diets. Patients 1 to 11 were followed in the UMCG (but P8 and P9 were mainly followed by the Glycogen Storage Disease Program, Connecticut, USA), whereas patients 12 to 15 were followed by the CMHI, Warsaw, Poland. The 15 GSD IV patients originated from 12 families and included 11 males and four females. Median follow‐up was 12.6 years (range 3.3‐31.8). Patients 1 to 6, 12, and 13 were diagnosed by either enzymatic and/or Sanger sequencing methods, whereas in patients 7 to 11, 14, and 15, the diagnosis was confirmed by NGS. Four patients from different families underwent LT, among whom three male patients. Interestingly, in two of these families an attenuated phenotype was observed in affected siblings, in whom LT was not deemed necessary.

**TABLE 1:** Patient characteristics of 15 GSD IV patients

Fam	P	Gender	Age at presentation (months)	Age at diagnosis (months)	Current age (years)	*GBE1* allele 1	*GBE1* allele 2	Age at LT (months)	Clinical phenotype	Dietary treatment summary^a^
I	1	M	19	30	31.8	np^b^	np	44	*Liver*: HSM, hypoglycaemia, liver dysfunction and cirrhosis, portal hypertension, transaminase elevation *Neuromuscular*: ‐	DH. PE D1‐2. LEM, PE D3. CNGDF, PE
II	2^c^	M	13	18	12.6	c.760A > G	c.2081 T > A	np	*Liver*: FI, fibrosis, FTT, HM, hypoglycaemia, transaminase elevation. *Neuromuscular*: hypotonia, mild developmental delay.	DH. PE D1. LEM, PE D2‐3.CNGDF, PE D4‐5. LEM, PE D5. PE
	3	M	None	72	13.7	c.760A > G	c.2081 T > A	np	*Liver*: ‐ *Neuromuscular*: mild exercise intolerance	D1‐2. ^d^
III	4^c^	M	27	34	12.7	c.691 + 2 T > P	c.176 T > C	37	*Liver*: FTT, HSM, liver cirrhosis, liver dysfunction, portal hypertension, transaminase elevation. *Neuromuscular*: ‐	D1‐3. LEM, UCCS, PE.
	5	F	None	50	14.0	c.691 + 2 T > P	c.176 T > C	np	*Liver*: FTT, FI, liver cirrhosis, portal hypertension, transaminase elevation. *Neuromuscular*: ‐	D1‐2.LEM, PE, UCCS D3‐4. LEM, UCCS
IV	6	M	0	27	12.7	c.1787G > A	c.1883A > G	33	*Liver*: FTT, HSM, hypoglycaemia, liver cirrhosis, liver dysfunction, portal hypertension. *Neuromuscular*: delayed motor development, hypotonia, muscle atrophy.	DH. PE D1‐2.CNGDF, PE
V	7	M	21	25	5.9	c.691 T + 2 T > C	c.760A > G	np	*Liver*: FI, hypoglycaemia *Neuromuscular*: delayed motor development, hypotonia, muscle pain.	D1‐3. LEM, UCCS, PE
VI	8^c^	M	30	36	6.1	c.986A > C	c.1106 + 5G > A	np	*Liver*: HM, liver bridging fibrosis, liver dysfunction *Neuromuscular*: hypotonia.	D1‐3. LEM, UCCS, PE
	9	M	None	9	3.3	c.986A > C	c.1106 + 5G > A	np	*Liver*: HM, transaminase elevation. *Neuromuscular*: ‐	D1‐3. LEM, UCCS, PE
VII	10	F	0	3	3.7	c.691 T + 2 T > C	c.1883A > G	np	*Liver*: FI, HK, hypoglycaemia. *Neuromuscular*: arthrogryposis, hypotonia.	D1‐2. LEM, UCCS, PE
VIII	11	F	0	33	5.5	c.1571G > A	c.1456_1458delInsAGT	np	*Liver*: HK, hypoglycaemia, liver dysfunction, transaminase elevation. *Neuromuscular*: arthrogryposis, hypotonia.	DH. CNGDF, PE D1. CNGDF, PE
IX	12	F	0	22	7^e^	c.263G > A	c.1621A > T	22	*Liver*: FTT, HSM, liver cirrhosis, liver dysfunction, portal hypertension, transaminase elevation. *Neuromuscular*: delayed motor development, hypotonia, muscle atrophy. *Other*: hypertrophic cardiomyopathy.	D1. None
X	13	M	9	26	19	IVS5 + 2 T > C	c.2081 T > A	np	*Liver*: HM, liver fibrosis, transaminase elevation. *Neuromuscular*: ‐	D1. PE
XI	14	M	5	288	26	c.2056 T > C	c.1570C > T	np	*Liver*: ‐ *Neuromuscular*: ‐ *Other*: mitral insufficiency, multiform ventricular arrhythmia.	D1. None
XII	15	M	0	30	5	c.691 + 2 T > C	c.785G > A	np	*Liver*: FTT, fibrosis, HSM, liver dysfunction, portal hypertension, transaminase elevation. *Neuromuscular*: arthrogryposis, delayed motor development.	D1. PE

^a^ Dietary restriction of mono‐ and disaccharides.

^b^ Enzyme activity in leucocytes 18 nmol/min/mg (ref: 180‐600 nmol/min/mg).

^c^ Index patient.

^d^ Diet history (DH) describes the diet before referral to our respective centres. D1 is the first prescribed diet after referral. Additional diets with changes regarding modality or composition of the diet are numbered in order and are further elaborated in the File S1.

^e^ This patient died at the age of 7; P7 was previously reported elsewhere,^13^ P12‐14 were previously reported elsewhere.^14^ P1‐P11 were followed in the UMCG (but P8 and P9 were mainly followed by the Glycogen Storage Disease Program, Connecticut, USA), whereas P12‐P15 were followed by The Childrens' Memorial Health Institute, Warsaw, Poland.

Abbreviations: CNGDF, continuous nocturnal gastric dripfeeding; D, diet; DH, diet history; Fam, family; FI, fasting intolerance; FTT, failure to thrive; HM, hepatomegaly; HSM, hepatosplenomegaly; HK, hyperketosis; LEM, late evening meal; LT, liver transplant; np, not performed; P, patient; PE, protein enrichment; UCCS, uncooked cornstarch.

Table 2 summarized the follow‐up data of the effect of dietary treatment from the group of GSD IV patients with and without LT. Improvements can be seen in clinical, biochemical, and imaging data in both groups. Although the groups have a small sample size, median values for height‐for‐age (−1.1 to 0.2 SD), weight‐for‐age (−1.3 to 0.8 SD) and ALT (244 to 43 U/L) greatly improved in the GSD IV patients after initiation of dietary treatment. Interestingly, at presentation GSD IV patients with LT had more severe liver damage and liver function parameters, but nevertheless showed a significant improvement before LT was performed (median ALT improved from 244 to 134 U/L). However, ALT remained significantly higher in the transplanted GSD IV patients compared to the nontransplanted patients (134 U/L vs 31 IU/L).

**TABLE 2:** Follow‐up data of the effect of dietary treatment of 13 out of 15 GSD IV patients with and without liver transplant

Parameters	Unit	No LT, last value before DT	No LT, at last follow‐up	LT, last value before DT	LT, last value before LT
Number of patients		10/15	10/15	3/15	3/15
Age (mean, range)	years	5.4 (0.8‐24.0)	10.4 (3.4‐27.0)	2.7 (2.4‐2.8)	3.2 (2.7‐3.7)
Sex (M/F)		7 M; 3 F	7 M; 3 F	3 M	3 M
Clinical					
Height‐for‐age	SD	−1.4 (−2.3 to 1.1)^a^	0.6 (−1.5 to 1.3)^a^	−1.1 (−1.2 to −0.5)^a^	−0.1 (−0.3 to 0.2)^a^
Weight‐for‐age	SD	−1.4 (−2.9 to 1.6)^a^	1.2 (0.6‐1.8)^a^	−1.3 (−1.7 to 0.1)^a^	0.0 (−0.9 to 0.8)^a^
Biochemical (median, range)					
AST	U/L	216 (32‐705)^a^ ^,^ ^b^	34 (23‐96)^a^ ^,^ ^b^	705 (388‐886)^a^ ^,^ ^b^	223 (183‐317)^a^ ^,^ ^b^
ALT	U/L	177 (14‐389)	31 (17‐113)^b^	244 (151‐339)	134 (73‐193)^b^
GGT	U/L	75 (9‐126)	14 (7‐44)^b^	104 (96‐126)	78 (63‐101)^b^
Bilirubin total	μmol/L	4 (3‐39)	7 (3‐10)^b^	27 (18‐39)	35 (19‐37)^b^
Bilirubin direct	μmol/L	2 (<1‐15)	—	15 (4‐17)	8 (4‐25)
Thrombocytes	10̂9/L^a^	150 (59‐240)	255 (120‐308)	90 (86‐97)	78 (61‐94)
Albumin	g/L	44 (35‐47)	46 (44‐47)	35 (29‐43)	35 (32‐44)
PT	Sec	12 (10.9‐16.1)	12.8 (12.1‐13.8)	15.8 (13.7‐17.8)	14.8 (14.7‐14.9)
CK	U/L	61 (42‐172)	122 (53‐224)	82 (23‐100)	103 (102‐104)
NT‐pro‐BNP	Ng/L	56 (29‐100)	24 (18‐29)	—	—
Imaging					
Hepatomegaly		2 Yes; 3 No; 5 Nm	0 Yes; 6 No; 4 Nm	3/3 Yes	3/3 Nm
Splenomegaly		1 Yes; 4 No; 5 Nm	2 Yes; 4 No; 4 Nm^b^	3/3 Yes	3/3 Nm^b^
Portal hypertension		0 Yes; 5 No; 5 Nm^b^	1 Yes; 5 No; 4 Nm	3/3 Yes^b^	1 Yes; 2 Nm

*Note*: Values per parameter are displayed as median and range. Data of P12 and P14 were excluded since no formal dietary treatment was prescribed.

^a^ Indicates a significant difference before and after initiation of dietary treatment.

^b^ Indicates a significant difference between patients with and without LT.

Abbreviations: ALT, alanine aminotransferase; AST, aspartate aminotransferase; CK, creatinine kinase; DT, dietary treatment; F, Female; GGT, Gamma‐glutamyl transferase; LT, liver transplant; M, Male; Nm, not measured; NT‐pro‐BNP, N‐terminal pro‐hormone brain natriuretic peptide; Nm, not measured; PT, prothrombin time; sec, seconds; SD, standard deviation.

All patients are currently alive apart from P12 who died from sepsis with pulmonary abscess and breathing difficulties at 7 years of age. The other three patient who received a LT (P1, P4, P6) have a follow‐up after LT of 28, 9.5 and 10 years, respectively, without extrahepatic manifestations.

Supplementary File S1 summarizes the detailed case histories of individual GSD IV patients including longitudinal information on the medical diet interventions, markers of metabolic control (ie, biometry, serum aminotransferases, glucose homeostasis, and ketones), liver function (ie, synthesis, bile flow and detoxification of protein, portal hypertension) and cardiac and/or neuromuscular involvement. In 13 out of 15 patients, medical diets were prescribed, including LEM (P1, P2, P4‐5, P7‐P10), UCCS supplementation (P4‐5, P7‐10), PE (P1‐P2, P4‐11, P13, P15), and CNGDF (P1, P2, P6, P11). P3 only received mono‐ and disaccharide restriction. Two patients did not receive a formal medical diet (P12, P14). Table 3 summarizes suggested monitoring and dietary treatment for GSD IV patients.

**TABLE 3:** Suggested monitoring and dietary treatment for GSD IV patients.

Recommendations for primary evaluation and monitoring: • Growth parameters (such as weight‐for‐age, height‐for‐age, weight‐for‐height) Symptoms and signs of: Fasting (in)tolerance (such as sympathicoadrenal response, proteolysis, hyperketosis, neuroglycopenia) Liver cirrhosis Portal hypertension (such as splenomegaly, oesophageal varices) Neuromuscular complications Cardiac complications Laboratory assessment: Blood glucose Blood lactate Uric acid Parameters for liver damage (ALT, AST) Parameters for liver function Synthesis (APTT, PT, INR, albumin, thrombocytes) Bile flow (total and direct bilirubin, GGT, AP) Detoxicifaction of protein (ammonia) Pre‐albumin Serum lipid profile (such as triglycerides, total cholesterol) Plasma CK Plasma NT‐pro‐BNP Urinary tetrasaccharide Abdominal doppler ultrasound (liver, spleen and portal veins) Cardiological assessment ECG Echocardiography At home selfmonitoring: Capillary glucose and 3‐hydroxybutyrate measurements with portable handdevices Continuous Glucose Monitoring Dietary treatment: Dietary treatment should be titrated based on the individual patient Consult a metabolic dietician Initiate dietary treatment in parallel with consulting the liver transplantation team Aim to prevent catabolism, glycogen accumulation and hyperammonemia Normoglycaemia, defined as the absence of preprandial signs of fasting intolerance or hypoglycemia (≤3.9 mmol/L or ≤70 mg/dl) in the absence of hyperglycemia Morning 3‐hydroxybutyrate concentrations in the normal range (< 0.3 mmol/L) Ensure adequate caloric intake Daytime frequent feeds (including complex carbohydrates, avoidance of mono‐ and disaccharides, high protein diet) Consider nocturnal management with bedtime snack, UCCS or CNGDF

Abbreviations: ALT, alanine aminotransferase; AST, aspartate aminotransferase; APTT, activated partial thromboplastin time; CNGDF, continuous nocturnal gastric drip feeding; PT, prothrombin time INR, International Normalized Ratio; GGT, gamma‐glutamyl transferase; AP, alkaline phosphatase; CK, creatinine kinase; NT‐pro‐BNP, N‐terminal pro hormone brain natriuretic peptide; ECG, electrocardiogram; UCCS, uncooked cornstarch.

**FIGURE 1:**
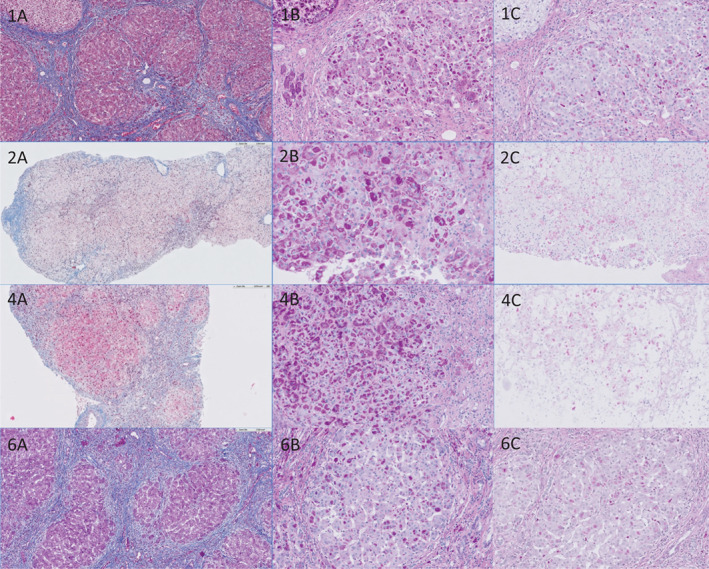
Histological staining of liver biopsies and explants of GSD IV patients. Histological staining of patients 1, 2, 4, and 6, respectively. Histology from P5 is described in Figure 2. The histology shown from P1 and P6 are explants and the histology from P2 and P4 are liver biopsies. A, Masson trichrome staining. B, PAS staining. C. PAS‐D staining. Two biopsies and all explants showed cirrhotic liver parenchyma with nodules hepatocytes surrounded with fibrotic septa. Variable sinusoidal and perivenular fibrosis was also present. Interface hepatitis is present in all biopsies and explants whereas lobular inflammation was mild in two explants (P1, P6) and one liver biopsy (P2). Lobular inflammation was absent in the remaining two biopsies (P4, P5) and explant (P4). All biopsies and explants showed similar mild to moderate portal lymphocytic inflammation. The liver biopsy and the explant of P4 had similar histological features. One liver biopsy (P2) showed septal fibrosis but no nodular architectural changes of the liver parenchyma. Mild perivenular fibrosis and sinusoidal fibrosis was also present. In the PAS staining of all biopsies and liver explants the eosinophilic inclusions were present. However, in all three explants some cirrhotic nodules were noticed composed of hepatocytes with abundant glycogen rich cytoplasm in the PAS staining with hardly any eosinophilic inclusions in both the PAS and PAS‐D staining. The amount of inclusions varied from nodule to nodule. The same pattern was seen in the PAS‐D slides. When compared with the PAS staining all biopsies showed partial resorption. PAS, periodic acid‐Schiff; PAS‐D, periodic acid‐Schiff after digestion

**FIGURE 2:**
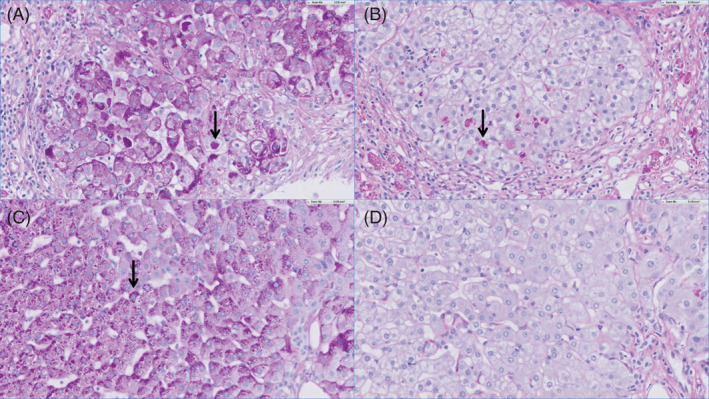
Typical and atypical inclusions in the liver biopsy of patient 5. A, PAS staining with typical inclusions. B, PAS‐D staining with typical inclusions. C, PAS staining with atypical inclusions. D, PAS‐D staining with atypical inclusions. The black arrow indicates an example of an inclusion. PAS, periodic acid‐Schiff; PAS‐D, periodic acid‐Schiff after digestion

Liver biopsies or explants and their histology descriptions were available from 8 out of 15 patients (P1‐2; P4‐P6, P8, P12, and P13). Liver biopsies from three patients (P2, P4, and P5) and three liver explants (patients P1, P4, and P6) were available for single investigator histological reassessment. A description of representative histological presentation is presented in Figure 1. However, no clear histological differences were demonstrated that could further explain the differences in clinical presentation between the patients. Interestingly, the recently described atypical histological characteristics with resorption of most inclusions of the PAS‐D staining^21^ could be seen in the liver biopsy of patient 5, although typical histological features were present in the same biopsy (Figure 2).

## 4 DISCUSSION

The prognosis for children diagnosed with GSD IV has traditionally been considered poor, and many patients have been referred immediately for LT at the time of diagnosis. In this report, successful management of this condition is described using medical diets, which aim to limit the accumulation of glycogen and to prevent catabolism. Medical treatment not only has delayed or prevented LT, but improved growth, fasting tolerance and normalization of serum aminotransferases also occurred.

While dietary management aimed at preventing glycogen storage is standard of care for the other hepatic forms of GSD, there is a paucity of literature on dietary treatment in GSD IV. Greene et al reported nutritional management in two GSD IV patients with asymptomatic fasting induced hypoglycaemia by 13 months of age. The treatment consisted of PE meals and UCCS with the goal of maintaining euglycaemia and adequate nutrient intake. The treatment improved hepatic size, serum transaminase values, prothrombin time and muscle strength. Goldstein et al reported on one 18 months old male patient who improved in growth and weight and had no deterioration of liver function on a high‐protein low‐carbohydrate diet before LT was performed 9 months later^22^. McConkie et al reported on four patients with the nonprogressive form of GSD IV.^8^ In three out of four of their patients, no unique dietary findings could be identified from their nutritional data, whereas in the fourth patient the nutritional data were not analyzed. Recently, Szymańska et al demonstrated the improvement of liver size, growth and liver function in one GSD IV patient after initiation of a relatively high protein diet and carbohydrate restriction.^17^

In GSD IV patients with progressive liver disease without LT, death from liver failure usually occurs by the age of 5 years. LT is considered the only treatment option in these patients. Therefore, selection and preparation of appropriate candidates for LT and timing of LT are complex and should parallel initiation of dietary treatment. This study reports a relatively long period of follow‐up without extrahepatic disease manifestations (28, 9.5, and 10 years, respectively) in three of our four transplanted patients (P1, P4, P6). According to existing literature, the prognosis is considered poor after LT because of risk for morbidity and mortality from extrahepatic manifestations, especially cardiomyopathy.^2^, ^5^, ^23^, ^24^ Out of 20 GSD IV patient reported in literature after LT, two required a second LT for unreported reasons, six died (four from sepsis, one from hepatic artery thrombosis, and one from cardiomyopathy.^5^ Interestingly, this group was composed of 17 boys and only three girls. To date, it is an enigma why some patients seem to be protected from a progressive liver cirrhosis (P3, P5) and what is the role of gender.

GSD IV patients have been phenotypically classified spanning a continuum of different subtypes.^25^ It is notable that hypoglycaemia has traditionally been deemed late manifestations in GSD IV patients, but in this study, fasting intolerance (evidenced by careful history taking, hypoglycaemia and/or ketosis) was documented in most of the patients without biochemical or radiological evidence of liver injury or hepatocellular dysfunction, but whom merely displayed a neuromuscular subtype (P6, P7, P10, and P11). We observed improved clinical (symptoms and signs) and biochemical outcomes after dietary interventions (Tables 1 and 2), but obviously, it is not clear if the improvement was due to prevention of abnormally formed glycogen accumulation or hyperketosis. Hepatic fibrosis and cirrhosis are also observed in GSD III, another GSD subtype in which abnormally formed glycogen is accumulating in the liver.^26^, ^27^ Catabolism evidenced by elevated 3‐hydroxybutyrate concentrations has been associated with hepatic fibrosis and development of cirrhosis in GSD IX (^28^). However, there is yet insufficient experimental or clinical evidence that hyperketosis and catabolism are independently and causally related to fibrosis or cirrhosis. Additional studies are warranted in experimental models for GSD IV to elucidate the pathogenesis of hepatic injury and hepatocellular dysfunction. To date, two naturally occurring animal models of GSD IV have been described; the American quarter horse^29^ and the Norwegian cat.^30^ These models have a severe phenotype and would be ideal for studying dietary strategies for this disorder. A mouse model for GSD IV also has been described with a slightly milder phenotype.^31^

Our study is biased by developments in health care for patients with ultra‐rare genetic diseases in the last decades. First, diagnostic procedures have changed from mainly clinical pattern recognition, subsequent enzymatic studies, *GBE1* Sanger sequencing toward a phenotype‐based NGS approach. This likely has shortened the diagnostic odyssey for patients and subsequent early diagnosis has driven questions about prognosis and management. Second, referrals and thereby inclusion for this study were influenced by the UMCG hosting both the national pediatric LT program and a centre of expertise for patients with liver GSD. This may have influenced the cohort as a whole toward GSD IV patients with a more progressive hepatic phenotype, in whom LT was considered at the time of referral. Additionally, Internet and social media empower patients, their families, and health care professionals in accessing expertise on this rare condition. Third, the study is biased by an impossibility to study natural progression of the GSD IV patients without dietary treatment. Last, other methodological limitations are the retrospective collection of data and the fact that adherence to the prescribed medical diet could not be formally assessed.

Evidence‐based or expert‐based guidelines for dietary management in GSD IV are not available. Based on the known enzymatic defect, the centres of expertise created dietary plans aimed at minimizing the formation of glycogen and preventing catabolism. Dietary treatment in GSD IV patients should be individualized and carefully titrated. This can be supported by home site monitoring of glucose, to maintain euglycaemia, to prevent fasting ketosis, and to ensure adequate nutrient intake. Hyperglycaemia should be avoided to minimize glycogen accumulation. Multidisciplinary monitoring includes balancing between traditional markers of metabolic control (ie, growth, liver size, serum aminotransferases, glucose homeostasis, lactate and ketones), liver function (ie, synthesis, bile flow and detoxification of protein) and symptoms and signs of portal hypertension, and cardiac and neuromuscular complications.

To conclude, this study demonstrates the potential of dietary management in a subset of GSD IV patients, as it should be considered in clinically stable patients prior to pursuing LT. This is particularly important as new treatments are being investigated for the hepatic glycogen storage diseases, including GSD IV, such as pharmacologic therapies,^32^ gene therapy,^33^ base editing,^34^ RNA inhibition,^35^ and mRNA therapy.^36^

## CONFLICT OF INTEREST

The authors have no conflicts of interest relevant to this article to disclose.

## AUTHOR CONTRIBUTIONS

Terry G. J. Derks initiated this project, was involved in study design, data collection, data analysis, wrote the first and final version of the manuscript. Patrick T. Ryan and David A. Weinstein were involved in study design, data collection, data analysis, and wrote the first and final manuscript. All other authors contributed to data collection and revised the manuscript for important intellectual content. All authors approved the final manuscript as submitted and agreed to be accountable for all aspects of the work. All authors confirm the absence of previous similar or simultaneous publications.

## ETHICS STATEMENT

The Medical Ethical Committee of the University Medical Center Groningen stated that the Medical Research Involving Human Subjects Act was not applicable and that official study approval by the Medical Ethical Committee was not required (METc 2019/119). The study was approved for waived consent as it concerned retrospective, anonymous data. In the United States, the data were collected as part of a natural history protocol with oversight from the Connecticut Children's IRB with signed consent from the parents (IRB# 17‐003). In Poland, in the Children's Memorial Health Institute the data were collected as part of a natural history protocol and according to this, no IRB's consent is required.

## Supporting information


**Appendix S1:** Supporting Information
Table S1 Detailed case histories and descriptions of dietary interventions in 15 GSD IV patients.
**Legend:** *The prescribed diet was initiated during this visit (Diet 1, etc.). The outcomes below the dietary intervention are measured at the visit or the last measurement before the visit. Diet history is the diet followed in the previous year by the patient. **As detected by abdominal imaging. ~ 1 time per 2 days 65 g UCCS.
**Abbreviations:** AP, alkaline phosphate; CK, creatinine kinase; CK‐MB, creatinine kinase isoenzyme muscle‐brain; CNGDF, continuous nocturnal gastric drip feeding; GGT, Gamma‐glutamyl transferase, LEM, late evening meal; LT, liver transplantation; NT‐pro‐BNP, N‐terminal pro‐hormone brain natriuretic peptide; PE, protein enrichment; UCCS, uncooked cornstarch; US, ultra sound.
