# Supplementary material for: The potential of dietary treatment in patients with glycogen storage disease type IV
Source: J Inherit Metab Dis. 2020 Dec 21;44(3):693–704. doi: 10.1002/jimd.12339 (PMC8246821; doi:10.1002/jimd.12339)
Supplement: Supplementary file 1 — Appendix S1: Supporting Information Table S1 Detailed case histories and descriptions of dietary interventions in 15 GSD IV patients. Legend: *The prescribed diet was initiated during this visit (Diet 1, etc.). The outcomes below the dietary intervention are measured at the visit or the last measurement before the visit. Diet history is the diet followed in the previous year by the patient. **As detected by abdominal imaging. ~ 1 time per 2 days 65 g UCCS. Abbreviations: AP, alkaline phosphate; CK, creatinine kinase; CK‐MB, creatinine kinase isoenzyme muscle‐brain; CNGDF, continuous nocturnal gastric drip feeding; GGT, Gamma‐glutamyl transferase, LEM, late evening meal; LT, liver transplantation; NT‐pro‐BNP, N‐terminal pro‐hormone brain natriuretic peptide; PE, protein enrichment; UCCS, uncooked cornstarch; US, ultra sound. [file JIMD-44-693-s001.docx]

**Supplementary file 1. Detailed case histories and descriptions of dietary interventions in 15 GSD IV patients.**

**Patient 1**

After a healthy pregnancy and delivery, patient was born after a full term with a birth weight of 3600 gram from a G3P2 mother. The medical history includes a pylorotomy for pyloric hypertrophia at four weeks of age and an adenotomy for nasal complaints, enlarged cervical lymph nodes and recurrent otitis at the age of nineteen months. The last procedure was complicated by a re-bleeding for which patient was admitted to the paediatric department. During the admission, he had a hepatomegaly (solid, 5-6 cm below right costal margin) and splenomegaly (solid, 2-3 cm below left costal margin), which was confirmed by ultrasound. Furthermore, he had delayed motor development (only crawling, no standing or walking at age nineteen months). Laboratory investigations found increased aminotransferases and bile flow abnormalities. A liver biopsy revealed micro and macro nodular liver cirrhosis with an increased collection of diastase-resistant, periodic acid-Schiff (PAS) positive material. The combination of hepatomegaly, liver cirrhosis at this age in combination with specific findings of liver biopsy suggested the diagnosis GSD IV, which was later confirmed by strongly decreased *GBE1* enzyme activity. At the age of 2.8 years, frequent meals were started on a maltodextrin base, protein enriched, and slightly limited in lactose, saccharose and fructose (diet 1). At age 3.5 the liver function and damage parameters had not improved and CNGDF was started (also on a maltodextrin base). At the time of LT 3 months later, the AST and ALT improved significantly (433 U/L to 183 U/L and 185 U/L to 134 U/L respectively). There was also an improvement of GGT (from 153 U/L to 101 U/L respectively). The main reason for LT was the remaining failure to thrive, in combination with the poor prognosis of GSD IV at that time.
 At age 31, 28 years after LT, the patient has not presented clinically with neuromuscular or cardiac involvement (CK at latest follow-up 182 U/L; NT-pro-BNP 20 ng/L).

**Supplementary Table 1. Dietary Treatment and Outcomes for Patient 1.**

| **Prescribed diet*** | | **Diet history** | **Diet 1** | **Diet 2** | **Diet 3** | **Pre LT** |
| --- | --- | --- | --- | --- | --- | --- |
| Parameter | Unit |  |  |  |  |  |
| Age | Years | 2.8 | 2.9 | 3.3 | 3.5 | 3.7 |
| **Dietary Treatment** | | PE | LEM, PE | LEM, PE | CNGDF, PE | CNGDF, PE |
| Total energy | kcal/day | 1509 | 1389 | 1972 | 2008 |  |
| Total protein | g/day | 42 | 37 | 49 | 54 |  |
|  | g/kg/day | 3.3 | 2.9 | 3.6 | 4.0 |  |
|  | % of total energy | 11% | 11% | 10% | 11% |  |
| Dietary protein | g/day | 42 | 37 | 49 | 54 |  |
|  | g/kg/day | 3.3 | 2.9 | 3.6 | 4.0 |  |
| Protein from supplements | g/day | 0 | 0 | 0 | 0 |  |
|  | g/kg/day | 0.0 | 0.0 | 0.0 | 0.0 |  |
| Total fat | g/day | 53 | 56 | 63 | 85 |  |
|  | % of total energy | 32% | 36% | 29% | 38% |  |
| Total carbohydrates | g/day | 220 | 184 | 297 | 261 |  |
|  | % of total energy | 58% | 53% | 60% | 52% |  |
| Complex carbohydrates | g/day |  |  |  |  |  |
|  | g/kg/day | 0.0 | 0.0 | 0.0 | 0.0 |  |
| **Biometry** | |  |  |  |  |  |
| Height | cm | 94.0 | 96.8 | 98.0 | 99.4 | 102 |
| Height-for-age | SD | -0.5 |  |  |  | -0,3 |
| Weight | kg | 12.9 | 12.9 | 13.6 | 13.6 | 15.2 |
| Weight-for-age | SD | -1.3 |  |  |  | -0.88 |
| Weight-for-height | SD | 1.4 |  |  |  | -0.97 |
| **Metabolic control** | |  |  |  |  |  |
| Glucose | mmol/L | 4.2 | 4.3 | - | - | 3.5 |
| Lactate | mmol/L | - | - | - | - | - |
| Uric acid | mmol/L | 0.28 | 0.15 | 0.22 | 0.28 | 0.23 |
| Triglyceride | mmol/L | 0.7 | 0.8 | 0.6 | - | 0.63 |
| Total cholesterol | mmol/L | 3.0 | 3.1 | 3.2 | 3.6 | 2.85 |
| Acetoacetate | mmol/L | - | - | - | - | - |
| Beta-OH-butyrate | mmol/L | - | - | - | - | - |
| **Cholestatic and liver parameters** | |  |  |  |  |  |
| AST | U/L | 388 | 285 | 390 | 433 | 183 |
| ALT | U/L | 151 | 134 | 176 | 185 | 134 |
| Ammonia | µmol/L |  | 20 | - | - | - |
| GGT | U/L | 96 | 99 | 148 | 153 | 101 |
| AP | U/L | 443 | 398 | 364 | 380 | 496 |
| Total bilirubin | µmol/L | 18 | 8 | 23 | 30 | 35 |
| Direct bilirubin | µmol/L | 4 | 2 | 4 | 11 | 4 |
| Thrombocytes | 10^9/L | - | 90 | 100 | 173 | 94 |
| APTT | Sec | 30 | - | - | - | - |
| PT | Sec | - | - | - | - | - |
| Albumin | g/L | 43 | 43 | 45 | 51 | 44 |
| Hepatomegaly** |  | Yes | - | - | - | - |
| Splenomegaly** |  | Yes | Yes | Yes | Yes | - |
| Portal hypertension |  | - | Yes | Yes | - | - |
| Ascites** |  | No | - | - | - | - |
| Cirrhosis |  | No | - | - | - | Yes |
| **Muscle involvement** | |  |  |  |  |  |
| CK | U/L | 23 | - | - | - | 41 |
| **Cardiac involvement** | |  |  |  |  |  |
| NT-pro-BNP | ng/L | - | - | - | - | - |
| ECG |  | - | - | - | - | - |
| US |  | - | - | - | - | - |

**Patient 2**

After a pregnancy complicated by maternal diabetes gravidarum and an uneventful delivery (birth weight 2200 grams, 39+3/7 weeks, Apgar 9/10), this boy presented with feeding problems, mild developmental delay, hypotonia and failure to thrive in the first year of life. At 13 months, he presented at the emergency department with fever and breathing difficulties. He was found to have hepatomegaly and increased serum aminotransferases for which he was referred to a paediatrician.

In the first 1.5 years of life, the patient experienced failure to thrive (height-for-age decreased from -0.6 to -2.3 SD; weight-for-age decreased from -0.4 to -2.9 SDS) and increased liver enzymes (ALT 389 U/l and AST 670 U/l), with only mild elevation of CK-total (270 U/l) and normal lipids. Liver synthesis (clotting studies, albumin), detoxification (ammonia) and bile flow (GGT, AP) functions remained normal. With clinical suspicion of GSD IV, a liver biopsy was performed, and liver histology demonstrated increased collection of diastase-resistant, periodic acid-Schiff (PAS) positive material and hepatocellular decay with fibrosis and early septum formation, but no cirrhosis yet. Afterwards, the diagnosis of GSD IV was enzymatically confirmed.

Before diagnosis, he was already on an energy enriched, high protein diet for the failure to thrive. At age 1.5 LEM was introduced. Two weeks later CNGDF was introduced via PEG-tube. At four years of age, both the motor and mental development had normalized. Over the course of the subsequent years, the diet was adjusted to the weight. Despite compliance issues, the biometry and serum aminotransferases improved significantly. Because the boy showed interest in removing the PEG-tube and the parents questioned whether the CNGDF could be replaced by a LEM, a controlled fasting study was performed at the age of 7.2 years. After a test meal of 65g UCCS and 15g protein powder and 12 hours of fasting, he displayed normoglycemia (4.2 mmol/L) with mildly increased ketones (beta-hydroxybutyrate plus acetoacetate were 0.76 mmol/L) and free fatty acids (1,104 µmol/l). In the subsequent years, the parents gradually stopped the LEM but maintained restriction of simple sugars, with improved compliance. Aminotransferases gradually normalized upon follow-up and by abdominal ultrasound, there were never signs of liver cirrhosis, portal hypertension or focal lesions. Muscle ultrasound and dynamometry did not display myopathy and no clear signs or symptoms of cardiomyopathy has been observed.

At age 12, imaging studies have not shown focal lesions or cirrhosis of the liver, no signs of portal hypertension, and the patient has not presented clinically with neuromuscular or cardiac involvement (CK-total at latest follow-up 113 U/L; NT-pro-BNP 52 ng/L).

**Supplementary Table 2. Dietary Treatment and Outcomes for Patient 2.**

| **Prescribed diet** | | **Diet history** | **Diet 1** | **Diet 2** | **Diet 3** | **Diet 4** | **Diet 5** | **Diet history** |
| --- | --- | --- | --- | --- | --- | --- | --- | --- |
| Parameter | Unit |  |  |  |  |  |  |  |
| Age | Years | 1.5 | 1.5 | 1.6 | 3.4 | 7.1 | 8.1 | 12.3 |
| **Dietary Treatment** | | PE | LEM, PE | CNGDF, PE | CNGDF, PE | LEM, PE | LEM, PE | PE |
| Total energy | kcal/day | 1533 | 1615 | 1459 | 2040 | 1645 | 1698 | 1506 |
| Total protein | g/day | 62 | 67 | 54 | 70 | 77 | 84 | 108 |
|  | g/kg/day | 7.2 | 7.8 | 6.1 | 4.6 | 2.8 | 3.1 | 2.3 |
|  | % of total energy | 16% | 17% | 15% | 14% | 19% | 20% | 29% |
| Dietary protein | g/day | 62 | 67 | 54 | 70 | 52 | 84 | 108 |
|  | g/kg/day | 7.2 | 7.8 | 6.1 | 4.6 | 1.9 | 3.1 | 2.3 |
| Protein from supplements | g/day | 0 | 0 | 0 | 0 | 15 | 0 | 0 |
|  | g/kg/day | 0,0 | 0,0 | 0,0 | 0,0 | 0,5 | 0,0 | 0,0 |
| Total fat | g/day | 80 | 77 | 62 | 89 | 49 | 61 | 55 |
|  | % of total energy | 47% | 43% | 38% | 39% | 27% | 32% | 33% |
| Total carbohydrates | g/day | 139 | 161 | 170 | 244 | 224 | 197 | 136 |
|  | % of total energy | 36% | 40% | 47% | 48% | 54% | 46% | 36% |
| Complex carbohydrates | g/day |  |  |  |  | 65 | 32.5~ |  |
|  | g/kg/day | 0.0 | 0,0 | 0.0 | 0.0 | 2.3 | 1.2 | 0.0 |
| **Biometry** | |  |  |  |  |  |  |  |
| Height | cm | 77.0 | - | 77.0 | 99.0 | 126.7 | 132.5 | 154.2 |
| Height-for-age | SD | -1.9 | - | -2.3 | -0.5 | 0.0 | -0.1 | -0.4 |
| Weight | kg | 8.6 | 8.6 | 8.9 | 15.3 | 28.0 | 27.2 | 47.8 |
| Weight-for-age | SD | -2.9 | - | -2.8 | -0.5 | 1.2 | 0.2 | 1.0 |
| Weight-for-height | SD | -2.2 | - | -1.7 | -0.2 | 1.4 | -0.1 | 1.4 |
| **Metabolic control** | |  |  |  |  |  |  |  |
| Glucose | mmol/L | 6.8 | - | - | - | 5.4 | 5.4 | 4.6 |
| Lactate | mmol/L | - | - | - | - | - | - | 1.1 |
| Uric acid | mmol/L | 0.21 | - | - | - | - | - | 0.20 |
| Triglyceride | mmol/L | 1.0 | - | - | 0.5 | - | 0.4 | 1.1 |
| Total cholesterol | mmol/L | 4.4 | - | - | 3.1 | - | 3.6 | 4.0 |
| Acetoacetate | mmol/L | - | - | - | - | - | - | - |
| Beta-OH-butyrate | mmol/L | - | - | - | - | - | - | - |
| **Cholestatic and liver parameters** | |  |  |  |  |  |  |  |
| AST | U/L | 670 | - | - | 85 | - | 64 | 38 |
| ALT | U/L | 389 | - | - | 107 | - | 98 | 43 |
| Ammonia | µmol/L | - | - | - | - | - | - | - |
| GGT | U/L | 110 | - | - | 20 | - | 18 | 15 |
| AP | U/L | 228 | - | - | 174 | - | 155 | 178 |
| Total bilirubin | µmol/L | 3 | - | - | 3 | - | <3 | 3 |
| Direct bilirubin | µmol/L | <1 | - | - | <1 | - | 1 | - |
| Thrombocytes | *10^9/L | 240 | - | - | 353 | 345 | - | - |
| APTT | sec | 28 | - | - | 27 | - | 26 | 36 |
| PT | sec | 11 | - | - | 11 | - | 11 | 12 |
| Albumin | g/L | 45 | - | - | 47 | - | 45 | - |
| Hepatomegaly** |  | No | - | - | No | No | No | No |
| Splenomegaly** |  | No | - | - | No | No | No | No |
| Portal hypertension |  | No | - | - | No | No | No | No |
| Ascites** |  | No | - | - | No | No | No | No |
| Cirrhosis** |  | No | - | - | No | No | No | No |
| **Muscle involvement** | |  |  |  |  |  |  |  |
| CK | U/L | 67 | - | - | - | - | 85 | 115 |
| **Cardiac involvement** | |  |  |  |  |  |  |  |
| NT-pro-BNP | ng/L | - | - | - | - | - | - | 18 |
| ECG |  | - | - | - | - | - | - | No |
| US |  | - | - | - | - | - | - | - |

**Patient 3**

In this patient, GSD type IV was enzymatically and genetically confirmed after clinical ascertainment of his younger brother (P2).

For him, the parents never received a formal dietary prescription, the dietary information was obtained by history taking. The parents restricted simple sugars like for his brother. The diet had been slightly protein enriched because of low normal pre-albumin. He has not displayed any clinical (growth, liver size, neuromuscular) or biochemical abnormalities (aminotransferases always below upper limit of normal, normal lipids and liver function tests) and he is closely monitored.

At the age of 13.5 years, he is developing normally. There are no symptoms or signs of fasting intolerance, or dysfunction of the liver, skeletal muscle or heart. He plays hockey and his parents observe decreased exercise tolerance compared to his team members.

**Supplementary Table 3. Dietary Treatment and Outcomes for Patient 3.**

| **Prescribed Diet** |  | **Diet 1** | **Diet 2** |
| --- | --- | --- | --- |
| Parameter | Unit |  |  |
| Age | years | 10.2 | 13.5 |
| **Dietary Treatment** | | None | None |
| Total energy | kcal/day | 1566 | 1699 |
| Total protein | g/day | 46.5 | 74.2 |
|  | g/kg/day | Unknown | 1.2 |
|  | % of total energy | 12% | 17% |
| Dietary protein | g/day | 46.5 | 74.2 |
|  | g/kg/day | Unknown | 1.2 |
| Protein from supplements | g/day | 0.0 | 0.0 |
|  | g/kg/day | 0.0 | 0.0 |
| Total fat | g/day | 49.8 | 70.8 |
|  | % of total energy | 29% | 38% |
| Total carbohydrates | g/day | 226 | 182.8 |
|  | % of total energy | 58% | 43% |
| Complex carbohydrates | g/day | 0.0 | 0.0 |
|  | g/kg/day | 0.0 | 0.0 |
| **Biometry** | |  |  |
| Height | cm | - | 169.7 |
| Height-for-age | SD | - | 0.6 |
| Weight | kg | - | 60.0 |
| Weight-for-age | SD | - | 1.4 |
| Weight-for-height | SD | - | 1.0 |
| **Metabolic control** | |  |  |
| Glucose | mmol/L | 5.2 | - |
| Lactate | mmol/L | 1.8 | - |
| Uric acid | mmol/L | 0.14 | - |
| Triglyceride | mmol/L | 0.4 | - |
| Total cholesterol | mmol/L | 4.1 | - |
| Acetoacetate | mmol/L | 0.02 | - |
| Beta-OH-butyrate | mmol/L | 0.01 | - |
| **Cholestatic and liver parameters** | |  |  |
| AST | U/L | 32 | - |
| ALT | U/L | 14 | - |
| Ammonia | µmol/L | 30 | - |
| GGT | U/L | 9 | 13 |
| AP | U/L | 190 | 187 |
| Total bilirubin | µmol/L | 3 | 3 |
| Direct bilirubin | µmol/L | 2 | - |
| Thrombocytes | 10^9/L | - | - |
| APTT | sec | 31 | 36 |
| PT | sec | 11 | 12 |
| Albumin | g/L | 45 | - |
| Hepatomegaly** |  | No | No |
| Splenomegaly** |  | No | No |
| Portal hypertension |  | No | No |
| Ascites** |  | No | No |
| Cirrhosis** |  | No | No |
| **Muscle involvement** | |  |  |
| CK | U/L | 111 | - |
| **Cardiac involvement** | |  |  |
| NT-pro-BNP | ng/L | 29 | - |
| ECG |  | - | No |
| US |  | No | No |

**Patient 4**

After an uneventful pregnancy and delivery, this patient was referred at the age of 27 months with recurrent respiratory tract infections, failure to thrive, protruding abdomen due to hepatosplenomegaly, associated with anaemia and thrombopenia. A liver biopsy was taken at age of 2.8 years and microscopically demonstrated signs of liver cirrhosis and polyglucosan inclusions, after which the diagnosis GSD IV was confirmed enzymatically. At that time, AST and ALT were strongly increased (576 and 197 U/L respectively), and cholestatic and bile flow parameters were also increased. An endoscopy revealed three grade 1 oesophageal varices.

At the age of 2.8 a diet was started with frequent meals and a late evening meal with 1 g/kg UCCS. The amount of total protein was reduced to 3.2 g/kg/day because of high ammonia levels. After only three months, AST and ALT decreased to 223 and 73 U/L respectively, and GGT decreased from 134 to 79 U/L, but liver functions of synthesis (clotting), bile flow (AP), detoxification (ammonia) remained abnormal. Abdominal ultrasound and doppler studies showed hepatosplenomegaly and signs of portal hypertension, without ascites.

At age of 3.1 years, the patient underwent LT. The cirrhotic liver abnormalities had not improved microscopically. Upon follow-up, there is no dysfunction of the liver, skeletal muscle (CK-total 57 U/L) or heart (NT-pro-BNP 49 ng/L). He is currently an athletics national champion in his age category.

**Supplementary Table 4. Dietary Treatment and Outcomes for Patient 4.**

| **Prescribed Diet** |  | **Diet 1** | **Diet 2** | **Diet 3/Pre LT** |
| --- | --- | --- | --- | --- |
| Parameter | Unit |  |  |  |
| Age | years | 2.8 | 2.8 | 3.1 |
| **Dietary Treatment** | | LEM, UCCS, PE | LEM, UCCS, PE | LEM, UCCS, PE |
| Total energy | kcal/day | 1850 | 1767 | 1892 |
| Total protein | g/day | 58 | 47 | 45 |
|  | g/kg/day | 4.0 | 3.2 | 2.9 |
|  | % of total energy | 13% | 11% | 10% |
| Dietary protein | g/day | 58 | 47 | 45 |
|  | g/kg/day | 4.0 | 3.2 | 2.9 |
| Protein from supplements | g/day | 0.0 | 0.0 | 0.0 |
|  | g/kg/day | 0.0 | 0.0 | 0.0 |
| Total fat | g/day | Unknown | 45 | 62 |
|  | % of total energy | Unknown | 23% | 29% |
| Total carbohydrates | g/day | Unknown | 291 | 287 |
|  | % of total energy | Unknown | 66% | 61% |
| Complex carbohydrates | g/day | 15 | 20 | 25 |
|  | g/kg/day | 1.0 | 1.4 | 1.6 |
| **Biometry** | |  |  |  |
| Height | cm | 90.5 | 91.6 |  |
| Height-for-age | SD | -1.1 | -1.2 |  |
| Weight | kg | 14.6 | 14.5 | 15.4 |
| Weight-for-age | SD | 0.1 | -0.2 | 0.0 |
| Weight-for-Height | SD | 1.1 | 0.8 |  |
| ***Metabolic control*** | |  |  |  |
| Glucose | mmol/L | 5.0 | 8.0 | - |
| Lactate | mmol/L | - | - | - |
| Uric acid | mmol/L | 0.07 | - | - |
| Triglyceride | mmol/L | 1.2 |  | - |
| Total cholesterol | mmol/L | 4.6 |  | - |
| Acetoacetate | mmol/L | - | - | - |
| Beta-OH-butyrate | mmol/L | - | - | - |
| **Cholestatic and liver parameters** | |  |  |  |
| AST | U/L | 705 | 576 | 223 |
| ALT | U/L | 244 | 197 | 73 |
| Ammonia | µmol/L | - | - | - |
| GGT | U/L | 126 | 134 | 79 |
| AP | U/L | 340 | 361 | 246 |
| Total bilirubin | µmol/L | 39 | 45 | 37 |
| Direct bilirubin | µmol/L | 15 | 23 | 25 |
| Thrombocytes | 10^9/L | 9 | 97 | 61 |
| APTT | sec | 34 | 35 | 32 |
| PT | sec | 18 | 16 | 15 |
| Albumin | g/L | 35 | 35 | 32 |
| Hepatomegaly** |  | Yes | - | - |
| Splenomegaly** |  | Yes | - | - |
| Portal hypertension** |  | Yes | Yes | Yes |
| Ascites** |  | No | - | - |
| Cirrhosis |  | Yes** | - | Yes |
| **Muscle involvement** | |  |  |  |
| CK | U/L | 100 | - | 104 |
| **Cardiac involvement** | |  |  |  |
| NT-pro-BNP | ng/L | - | - | - |
| ECG |  | - | - | - |
| US |  | - | - | - |

**Patient 5**

In this patient, GSD type IV was enzymatically and genetically confirmed after clinical ascertainment of her younger brother (P4). The liver biopsy demonstrated signs of cirrhotic liver parenchymal tissue with diastase-resistant, periodic acid-Schiff (PAS) positive material in a part of the hepatocytes and septal macrophages. Patient 5 had already been followed by a paediatrician for failure to thrive. Before initiation of dietary treatment, the height-for age and weight-for-age were -0.8 and -0.3 SD respectively. Besides mildly elevated serum aminotransferases (AST 79 U/L; ALT 64 U/L), liver synthesis (clotting studies, albumin), detoxification (ammonia) and bile flow (GGT, AP) functions remained normal. An endoscopy revealed one grade 1 distal oesophageal varix. The patient started on a dietary regime at age 4.2 consisting of frequent meals, LEM of 1.2 g/kg/day of UCCS, and protein enriched of 3.8 g/kg/day. In parallel, since the liver was cirrhotic, LT was considered. However, since liver function improved after initiation of diet therapy and she did not have serious complications from liver cirrhosis, the patient did not receive an active waiting list status. In the following years the diet was adjusted based on weight and the protein intake was naturally restricted. Based on a relatively low pre-albumin (0.22 µmol/L), a protein intake around 2g/kg/day was advised. The biometry of the patient improved while the serum aminotransferases normalized. The liver microscopy findings were milder than in her brother.

At age 14, she is performing athletics at a national level like her brother without any signs of cardiac or muscle involvement (CK-total 38 U/L; NT-pro-BNP 18 ng/L). By ultrasound and doppler studies, hepatosplenomegaly persisted with normal portal flow velocities. The oesophageal varix decreased in size and is carefully monitored.

**Supplementary Table 5. Dietary Treatment and Outcomes for Patient 5.**

| **Prescribed diet** |  | **Diet 1** | **Diet 2** | **Diet history** | **Diet history** |
| --- | --- | --- | --- | --- | --- |
| **Parameter** | **Unit** |  |  |  |  |
| Age | years | 4.2 | 4.5 | 12.9 | 13.8 |
| **Dietary Treatment** |  | LEM, UCCS, PE | LEM, UCCS, PE | LEM, UCCS | LEM |
| Total energy | kcal/day | 1564 | 1458 | 2186 | - |
| Total protein | g/day | 63 | 69 | 92,3 | - |
|  | g/kg/day | 3.8 | 3.7 | 1.7 | - |
|  | % of total energy | 16% | 19% | 17% | - |
| Dietary protein | g/day | 63 | 59 | 92.3 | - |
|  | g/kg/day | 3.8 | 3.1 | 1.7 | - |
| Protein from supplements | g/day | 0.0 | 10 | 0.0 | - |
|  | g/kg/day | 0.0 | 0.5 | 0.0 | - |
| Total fat | g/day | 65 | 46 | 75.4 | - |
|  | % of total energy | 37% | 28% | 31% | - |
| Total carbohydrates | g/day | 179 | 190 | 278.5 | - |
|  | % of total energy | 46% | 52% | 51% | - |
| Complex carbohydrates | g/day | 20 | 30 | 50 | - |
|  | g/kg/day | 1.2 | 1.6 | 0.9 | - |
| **Biometry** |  |  |  |  |  |
| Height | cm | 102.6 | 106.0 | 166.4 | 168.0 |
| Height-for-age | SD | -0.8 | -0.6 | 0.9 | 0.6 |
| Weight | kg | 16.6 | 18.9 | 54.4 | 56.0 |
| Weight-for-age | SD | -0.3 | 0.4 | 0.9 | 0.6 |
| Weight-for height | SD | 0.3 | 1.1 | 0.2 | 0.2 |
| **Metabolic control** |  |  |  |  |  |
| Glucose | mmol/L | 4.9 | 5.0 | 4.6 | - |
| Lactate | mmol/L | - | - | 0.9 | - |
| Uric acid | mmol/L | 0.17 | - | 0.20 | - |
| Triglyceride | mmol/L | 0.9 | 0.5 | 0.6 | - |
| Total cholesterol | mmol/L | 2.8 | 3.1 | 3.0 | - |
| Acetoacetate | mmol/L | - | - | 0.02 | - |
| Beta-OH-butyrate | mmol/L | - | - | 0.00 | - |
| **Cholestatic and liver parameters** |  |  |  |  |  |
| AST | U/L | 79 | 75 | 35 | 30 |
| ALT | U/L | 64 | 101 | 38 | 36 |
| Ammonia | µmol/L | - | - | 16 | <10 |
| GGT | U/L | 78 | 69 | 30 | 34 |
| AP | U/L | 237 | 221 | 194 | 127 |
| Total bilirubin | µmol/L | 9 | 4 | 8 | 7 |
| Direct bilirubin | µmol/L | 3 | <1 | 2 | - |
| Thrombocytes | 10^9/L | 59 | 102 | 113 | 120 |
| APTT | sec | 28 | 29 | 34 | 34 |
| PT | sec | 12 | 12 | 11 | 14 |
| Albumin | g/L | 41 | 44 | 47 | 46 |
| Hepatomegaly** |  | - | - | No | No |
| Splenomegaly** |  | - | - | Yes | Yes |
| Portal hypertension |  | Yes | Yes | Yes | Yes |
| Ascites** |  | - | - | No | No |
| Cirrhosis |  | - | Yes | No** | No** |
| **Muscle involvement** |  |  |  |  |  |
| CK | U/L | 42 | - | 38 | - |
| **Cardiac involvement** |  |  |  |  |  |
| NT-pro-BNP | ng/L | - | - | 18 | - |
| ECG |  | - | - | - | - |
| US |  | - | - | - | - |

**Patient 6**

During the pregnancy, the mother of patient 6 developed HELLP syndrome. He was born prematurely after a gestational age of 30+4 weeks with a birth weight of 1450 grams. From birth onwards, he displayed hypotonia and delayed motor developmental with severe atrophic muscles in shoulders, hands and feet. Initially, these symptoms and signs were attributed to the prematurity.

The diagnosis GSD IV was made at age 2.3, and a month later, the patient had hematemesis as a result of three oesophageal varices for which he received banded ligation. Upon admission to the paediatric department, the patient had failure to thrive, hypoglycaemia (2.9 mmol/L), and severely elevated aminotransferases (AST 886 U/L; ALT 339 U/L), bile flow (GGT 104 U/L) and synthesis (albumin 29 g/L) abnormalities.

A diet was started based on frequent meals and CNGDF on a basis of maltodextrin and later energy enriched heparon, less enriched with protein because of hyperammonaemia (2.8 g/kg/day). In the next three months, the biometry improved significantly, the serum aminotransferases decreased by 50% and the hypoglycaemia disappeared. In parallel, his liver ultrasound displayed signs of cirrhosis (bumpy structure) and portal hypertension (collaterals in liver and spleen, ascites), with antegrade flow in the portal vein. He was approved for a LT, which he received at age 2.8. Liver histology demonstrated cirrhotic liver parenchymal tissue with diastase-resistant, periodic acid-Schiff (PAS) positive material.

At age 12, he is necessitating a walker for mobilisation, although CK levels remained normal (52 U/L). He did not develop signs of cardiac involvement (NT-pro-BNP 19 ng/L).

**Supplementary Table 6. Dietary Treatment and Outcomes for Patient 6.**

| **Prescribed diet** |  |  | **Diet 1** | **Diet 2** |
| --- | --- | --- | --- | --- |
| **Parameter** | **Unit** |  |  |  |
| Age | years | 2.4 | 2.4 | 2.7 |
| **Dietary Treatment** |  | PE | CNGDF, PE | CNGDF, PE |
| Total energy | kcal/day | 1249 | 1292 | 1194 |
| Total protein | g/day | 44 | 33 | 28 |
|  | g/kg/day | 3.8 | 2.8 | 1.8 |
|  | % of total energy | 14% | 10% | 9% |
| Dietary protein | g/day | 44 | 33 | 28 |
|  | g/kg/day | 3.8 | 2.8 | 1.8 |
| Protein from supplements | g/day | 0 | 0 | 0 |
|  | g/kg/day | 0 | 0 | 0 |
| Total fat | g/day | 42 | 53 |  |
|  | % of total energy | 30% | 37% | 0% |
| Total carbohydrates | g/day | 171 | 170 |  |
|  | % of total energy | 55% | 53% | 0% |
| Complex carbohydrates | g/day | 0 | 0 | 0 |
|  | g/kg/day | 0 | 0 | 0 |
| **Biometry** |  |  |  |  |
| Height | cm | 88.6 | 88.6 | 96 |
| Height-for-age | SD | -1.2 | -1.2 | 0.2 |
| Weight | kg | 11.7 | 11.7 | 15.8 |
| Weight-for-age | SD | -1.7 | -1.7 | 0.8 |
| Weight-for-Height | SD | -1.4 | -1.4 | 1.0 |
| **Metabolic control** |  |  |  |  |
| Glucose | mmol/L | 2.9 | - | 7.2 |
| Lactate | mmol/L | - | - | - |
| Uric acid | mmol/L | 0.23 | - | 0.18 |
| Triglyceride | mmol/L | 1.1 | - | 0.5 |
| Total cholesterol | mmol/L | 3.5 | - | 2.4 |
| Acetoacetate | mmol/L | - | - | - |
| Beta-OH-butyrate | mmol/L | - | - | - |
| **Cholestatic and liver parameters** |  |  |  |  |
| AST | U/L | 886 | - | 317 |
| ALT | U/L | 339 | - | 193 |
| Ammonia | µmol/L | - | - | - |
| GGT | U/L | 104 | - | 63 |
| AP | U/L | 136 | - | 298 |
| Total bilirubin | µmol/L | 27 | - | 19 |
| Direct bilirubin | µmol/L | 17 | - | 8 |
| Thrombocytes | 10^9/L | 86 | - | - |
| APTT | sec | 29 | - | 33 |
| PT | sec | 14 | - | 15 |
| Albumin | g/L | 29 | - | 35 |
| Hepatomegaly** |  | Yes | Yes | - |
| Splenomegaly** |  | Yes | - | - |
| Portal hypertension |  | Yes | Yes | - |
| Ascites** |  | Yes | Yes | - |
| Cirrhosis |  | Yes | Yes | - |
| **Muscle involvement** |  |  |  |  |
| CK | U/L | 82 | - | 102 |
| **Cardiac involvement** |  |  |  |  |
| NT-pro-BNP | ng/L | - | - | - |
| ECG |  | - | - | - |
| US |  | - | - | - |

**Patient 7**

The case description until the age of three years is reported elsewhere (Schene ea. 2019). In brief, the mother of patient 7 developed hypertension. He was born by Caesarean section after a gestational age of 37+4 weeks with a birth weight of 2680 grams. He was breastfed the first three days and hypoglycaemia occurred in the transition phase. For evaluation of his hypotonia and delayed motor development (despite physiotherapy), he was referred to a paediatric neurologist at the age of 21 months. By the age of 2.1 years, he received the diagnosis of GSD IV by next generation sequencing panel (74 genes for congenital and distal myopathy). In addition to the *GBE1* variants, he is compound heterozygote for two variants in the *COL6A2* gene (myosclerosis myopathy; OMIM #255600). Since the age of 2.5 years, the parents have noted him trembling while waking up and at home fasted hypoglycemia episodes were noted incidentally (2.6, 2.6 and 3.3 mmol/L).

At the age of 2.9 years, he was referred to our liver GSD center of expertise for dietary evaluation. The dietary intervention included restriction of mono-and disaccharides (maltodextrin was stopped), a daily protein intake of 2.5 g/kg/d and a late evening meal with 2g/kg UCCS and protein supplement. No symptoms or signs of fasting intolerance or hypoglycaemia were observed with CGM, nor has there been morning ketosis, increase of serum aminotransferases, dysfunction of the liver or cardiac involvement. The parents reported more energy and stamina, and less muscle pain. He learned to cycle without training wheels and didn’t need a corset anymore. Nevertheless, some muscle pains persisted while being active, the parents recognized he is slow in running compared to peers and he calls himself ‘weak’ while walking. He has not shown signs of cardiac involvement (NT-pro-BNP 29 ng/L).

**Supplementary Table 7. Dietary Treatment and Outcomes for Patient 7.**

| **Prescribed diet** |  | **Diet 1** | **Diet 2** | **Diet 3** |
| --- | --- | --- | --- | --- |
| **Parameter** | **Unit** |  |  |  |
| Age | years | 2.9 | 4.0 | 5.2 |
| **Dietary treatment** | | LEM, UCCS, PE | LEM, UCCS, PE | LEM, UCCS, PE |
| Total energy | kcal/day |  |  | 1589 |
| Total protein | g/day | 36 | 46 | 78.9 |
|  | g/kg/day | 2.5 | 2.5 | 3.2 |
|  | % of total energy | - | - | 20% |
| Dietary protein | g/day | 36 | 46 | 53.9 |
|  | g/kg/day | 2.5 | 2.5 | 2.2 |
| Protein from supplements | g/day | 0 | 0 | 25 |
|  | g/kg/day | 0.0 | 0.0 | 1.0 |
| Total fat | g/day |  |  | 47.4 |
|  | % of total energy | - | - | 27% |
| Total carbohydrates | g/day |  |  | 205.8 |
|  | % of total energy | - | - | 52% |
| Complex carbohydrates | g/day | 30 | 40 | 50 |
|  | g/kg/day | 2.1 | 2.2 | 2.0 |
| **Biometry** | |  |  |  |
| Height | cm | 97.4 | 108.4 | 121.1 |
| Height-for-age | SD | 0.2 | 0.7 | 1.3 |
| Weight | kg | 14.4 | 18.2 | 24.6 |
| Weight-for-age | SD | -0.4 | 0.4 | 1.8 |
| Weight-for-Height | SD | -0.6 | 0.1 | 1.1 |
| **Metabolic control** | |  |  |  |
| Glucose | mmol/L | 5.1 | 5.8 | 5.5 |
| Lactate | mmol/L | 1.1 | 0.7 | 1.0 |
| Uric acid | mmol/L | 0.18 | 0.15 | 0.16 |
| Triglyceride | mmol/L | 0.9 | 1.0 | 1.0 |
| Total cholesterol | mmol/L | 4.0 | 3.8 | 4.3 |
| Acetoacetate | mmol/L | 0.03 | - | - |
| Beta-OH-butyrate | mmol/L | 0.01 | - | - |
| **Cholestatic and liver parameters** | |  |  |  |
| AST | U/L | 36 | 38 | 34 |
| ALT | U/L | 18 | 21 | 26 |
| Ammonia | µmol/L | 36 | - | - |
| GGT | U/L | 11 | - | - |
| AP | U/L | 315 | - | - |
| Total bilirubin | µmol/L | 4 | - | - |
| Direct bilirubin | µmol/L | - | - | - |
| Thrombocytes | 10^9/L | - | - | - |
| APTT | sec | 29 | 35 | - |
| PT | sec | 11 | - | - |
| Albumin | g/L | 44 | 48 | - |
| Hepatomegaly** |  | - | - | No |
| Splenomegaly** |  | - | - | No |
| Portal hypertension |  | - | - | No |
| Ascites** |  | - | - | No |
| Cirrhosis |  | - | - | No** |
| **Muscle involvement** | |  |  |  |
| CK | U/L | 172 | 212 | 181 |
| **Cardiac involvement** | |  |  |  |
| NT-pro-BNP | ng/L | 100 | 42 | 29 |
| ECG |  | - | - | No |
| US |  | - | - | No |

**Patient 8**

This male patient was born vaginally at a 40-week gestation without complications. His birth weight was 3910 grams and he was not hypoglycemic at birth. He breastfed vigorously and he seemed to meet all his developmental milestones until he was nearly 2.5 years old when his parents noticed that he was lying around more and was not as active as other children his age. At 3 years of age, due to hepatomegaly, fatigue and transaminase elevation (AST 296 U/L, ALT 281 U/L, and GGT 101 U/L), he underwent a liver biopsy in which the morphologic findings are highly suggestive of GSD IV. Trichrome and reticulin stains demonstrated presence of bridging fibrosis with regenerative nodule formation. The portal tracts contained numerous plump histiocytes with lightly eosinophilic intracytoplasmic deposits. The morphologic findings were deemed consistent with GSD IV, and the diagnosis was confirmed after a next generation sequencing GSD panel found the *GBE1* variants (c.986A>C and c.1108+5G>A). Of note, there was also a c.1499T>C variant of uncertain significance in the *PHKB* gene (glycogen storage disease type IXb; OMIM #261750).

Following the diagnosis, a low carbohydrate diet was prescription with strict restriction of simple sugars. A diet with 3 g/kg of protein was prescribed along with cornstarch supplementation. With the interventions, clinical and biochemical improvement were documented. His beta-OH-butyrate concentrations remained below 0.4 mmol/L, and he experienced normalization of his serum aminotransferases. His triglycerides were 0.80 mmol/L, CK was 71 U/L and prealbumin was 17 mg/dL. His height improved from -0.19 SD to +0.3 SD and his weight improved from -0.13 SD to +0.6 SD.

**Supplementary Table 8. Dietary Treatment and Outcomes for Patient 8.**

| **Prescribed diet** |  | **Diet 1** | **Diet 2** | **Diet 3** |
| --- | --- | --- | --- | --- |
| **Parameter** | **Unit** |  |  |  |
| Age | years | 3.0 | 5.1 | 6.3 |
| **Dietary treatment** | | | | |
| **Type of dietary treatment** |  | LEM, UCCS, PE | LEM, UCCS, PE | LEM, UCCS, PE |
| Total energy | kcal/day |  | 2201 | 2375 |
| Total protein | g/day |  | 174.9 | 195.4 |
|  | g/kg/day |  | 8.7 |  |
|  | % of total energy |  | 32.7 | 34.5 |
| Dietary protein | g/day |  | 86.9 | 91.4 |
|  | g/kg/day |  | 4.3 |  |
| Protein from supplements | g/day | 62 | 88 | 104 |
|  | g/kg/day |  | 4.4 |  |
| Total fat | g/day |  | 54 | 89.1 |
|  | % of total energy |  | 22 | 33.3 |
| Total carbohydrates | g/day |  | 242.9 | 186.9 |
|  | % of total energy |  | 44 | 31 |
| Complex carbohydrates | g/day | 82 | 99 | 114 |
|  | g/kg/day |  | 4.9 |  |
| **Biometry** | | | | |
| Height | cm | 94.2 | 110.7 | - |
| Height-for-age | SD | -0.19 | 0.3 | - |
| Weight | kg | 14.7 | 20.1 | - |
| Weight-for-age | SD | -0.13 | 0.60 | - |
| Weight-for-Height | SD | -0.02 | 0.75 | - |
| **Metabolic control** | | | | |
| Glucose | mmol/L | - | 4.8 | - |
| Lactate | mmol/L | - | 1.0 | - |
| Uric acid | mmol/L | - | 0.13 | 0.14 |
| Triglyceride | mmol/L | - | 0.80 | 0.58 |
| Total cholesterol | mmol/L | - | 3.4 | 3.5 |
| Acetoacetate | mmol/L | - | - | - |
| Beta-OH-butyrate | mmol/L | - | 0.2 | - |
| **Cholestatic and liver parameters** | | | | |
| AST | U/L | 296 | 34 | 27 |
| ALT | U/L | 281 | 24 | 24 |
| Ammonia | µmol/L | - | - | - |
| GGT | U/L | 101 | - | 12 |
| AP | U/L | - | 313 | 337 |
| Total bilirubin | µmol/L | - | 5.1 | 3.4 |
| Direct bilirubin | µmol/L | - |  | - |
| Thrombocytes | 10^9/L | - | - | - |
| APTT | sec | - | - | - |
| PT | sec | - | - | - |
| Albumin | g/L | - | - | - |
| Hepatomegaly** |  | - | Yes | - |
| Splenomegaly** |  | - | No | - |
| Portal hypertension |  | - | - | - |
| Ascites** |  | - | - | - |
| Cirrhosis |  | No | - | - |
| **Muscle involvement** | | | | |
| CK | U/L | - | 71 | 119 |
| **Cardiac involvement** | |  |  |  |
| NT-pro-BNP | ng/L | - | - | - |
| ECG |  | - | - | - |
| US |  | - | - | - |

**Patient 9**

In this male patient was diagnosed after clinical ascertainment of his older brother (P8). This male was born vaginally after a 40-week gestation without complications. He weighed 4650 grams at birth and was not hypoglycemic during the transitional period. The pregnancy was complicated by hyperemesis gravidarum and the mother was on dexamethasone regularly. He breastfed vigorously and he has met all his developmental milestones to date. The same *GBE1* genotype and c.1499T>C variant in the *PHKB* gene as P8 were identified.

Following the diagnosis, a low carbohydrate diet was prescription with strict restriction of simple sugars. A diet with 3 g/kg of protein was prescribed along with cornstarch supplementation. With the interventions, clinical and biochemical improvement were documented.

His AST improved from 236 to 23 U/L, his ALT from 245 to 23 U/L and his GGT from 49 to 11 U/L. His triglycerides were 0.62 mmol/L. His CK was 121 U/L and his prealbumin was 17 mg/dL. His height improved from +1.07 SD to +1.74 SD and his weight increased from +1.42 SD to +2.73 SD.

**Supplementary Table 9. Dietary Treatment and Outcomes for Patient 9.**

| **Prescribed diet** |  | **Diet 1** | **Diet 2** | **Diet 3** |
| --- | --- | --- | --- | --- |
| **Parameter** | **Unit** |  |  |  |
| Age | years | 0.8 | 2.3 | 3.6 |
| **Dietary treatment** | | | | |
| Type of dietary treatment |  | LEM, UCCS, PE | LEM, UCCS, PE | LEM, UCCS, PE |
| Total energy | kcal/day |  | 2105 | 2071 |
| Total protein | g/day |  | 160.7 | 173 |
|  | g/kg/day |  | 9.0 |  |
|  | % of total energy |  | 31.1 | 35 |
| Dietary protein | g/day |  | 90.7 | 73 |
|  | g/kg/day |  | 5.1 |  |
| Protein from supplements | g/day | 33 | 70 | 100 |
|  | g/kg/day |  | 3.9 |  |
| Total fat | g/day |  | 54.9 | 77.2 |
|  | % of total energy |  | 23.4 | 33.3 |
| Total carbohydrates | g/day |  | 234.4 | 160.7 |
|  | % of total energy |  | 44.3 | 30.6 |
| Complex carbohydrates | g/day | 53 | 76.3 | 88 |
|  | g/kg/day |  | 4.3 |  |
| **Biometry** | | | | |
| Height | cm | 77.0 | 95.4 | - |
| Height-for-age | SD | 1.07 | 1.74 | - |
| Weight | kg | 10.2 | 17.9 | - |
| Weight-for-age | SD | 1.60 | 2.73 | - |
| Weight-for-Height | SD | -0.12 | 1.99 | - |
| **Metabolic control** | | | | |
| Glucose | mmol/L | 4.8 | 5.6 | 5.0 |
| Lactate | mmol/L | - | 1.0 | - |
| Uric acid | mmol/L | 0.26 | 0.22 | 0.14 |
| Triglyceride | mmol/L | 0.9 | 1.1 | 0.62 |
| Total cholesterol | mmol/L | 2.8 | 3.7 | 3.2 |
| Acetoacetate | mmol/L | - | - | - |
| Beta-OH-butyrate | mmol/L | - | 0.2 | - |
| **Cholestatic and liver parameters** | | | | |
| AST | U/L | 236 | 39 | 23 |
| ALT | U/L | 245 | 32 | 23 |
| Ammonia | µmol/L | - | - | - |
| GGT | U/L | 49 | - | 11 |
| AP | U/L | 283 | 244 | 245 |
| Total bilirubin | µmol/L | <3.5 | 10.3 | 6.8 |
| Direct bilirubin | µmol/L | - | - | - |
| Thrombocytes | 10^9/L | - | - | - |
| APTT | sec | - | - | - |
| PT | sec | - | - | - |
| Albumin | g/L | 42 | - | - |
| Hepatomegaly** |  | - | - | - |
| Splenomegaly** |  | - | - | - |
| Portal hypertension |  | - | - | - |
| Ascites** |  | - | - | - |
| Cirrhosis |  | - | - | - |
| **Muscle involvement** | | | | |
| CK | U/L | - | 121 | 125 |
| **Cardiac involvement** | | | | |
| NT-pro-BNP | ng/L | - | - | - |
| ECG |  | - | - | - |
| US |  | - | - | - |

**Patient 10**

She was born by Caesarean section at 38+5 weeks, birth weight 2940 grams, after an uncomplicated pregnancy. In retrospect, the mother may have experienced decreased fetal movements. She was floppy from the start with clear arthrogryposis. The diagnosis GSD IV was confirmed by exome sequencing at the age of three months.

Skeletal muscle was the main affected organ system. She couldn’t move towards independent sitting or standing, she moved with a wheelchair. At night, she received respiratory support by continuous positive airway pressure. Fasting intolerance was suggested by occasionally waking up during the night and asking for milk. At the age of twelve months the patient started with relative restriction of simple sugars and administration of UCCS at night. This diet was strictly followed from eighteen months onward. The parents were very engaged with searching information on the internet and social media groups. After visiting a patient organization conference and measuring fasting ketone levels until 3.8 mmol/l in the morning, they started extra protein in the diet.

At the age of 2.5 years, she was referred to our liver GSD center of expertise for dietary evaluation. At baseline, serum aminotransferases, liver function tests, CK and clinical cardiac investigations were normal. Abdominal ultrasound was normal. The dietary prescription included restriction of mono-and disaccharides, a daily protein intake of 3-4 g/kg/d and a LEM with UCCS and protein, titrated based on CGM-profiles and morning ketone levels > 1.0 mmol/l. A LEM with UCCS was continued and the total amount of protein was distributed more equally during the day (with a protein supplement for the night). After the diet modification, these parameters improved, she slept better and did not ask for milk anymore during the nights. At last follow-up liver parameters and cardiac parameters are unremarkable. Parents describe an improvement in motor function (i.e. more stability during sitting). Currently, she is following an intensive training program aimed at improving muscle and motor function.

**Supplementary Table 10. Dietary Treatment and Outcomes for Patient 10.**

| **Prescribed diet** |  | **Diet 1** | **Diet 2** | **Last follow-up** |
| --- | --- | --- | --- | --- |
| **Parameter** | **Unit** |  |  |  |
| Age | years | 2.8 | 2.9 | 3.4 |
| **Dietary treatment** |  |  |  |  |
| Type of dietary treatment |  | LEM, UCCS, PE | LEM, UCCS, PE | LEM, UCCS, PE |
| Total energy | kcal/day | 742 | 730 |  |
| Total protein | g/day | 66 | 61 |  |
|  | g/kg/day | 5.9 | 5.4 |  |
|  | % of total energy | 36% | 33% |  |
| Dietary protein | g/day | 34.6 | 34.6 |  |
|  | g/kg/day | 3.1 | 3.1 |  |
| Protein from supplements | g/day | 31.4 | 26.4 |  |
|  | g/kg/day | 2.8 | 2.4 |  |
| Total fat | g/day | 14 | 13.8 |  |
|  | % of total energy | 17% | 17% |  |
| Total carbohydrates | g/day | 83.6 | 86.2 |  |
|  | % of total energy | 45% | 47% |  |
| Complex carbohydrates | g/day | 10 | 15 |  |
|  | g/kg/day | 0.9 | 1.3 |  |
| **Biometry** |  |  |  |  |
| Height | cm | 87.0 | - |  |
| Height-for-age | SD | -2.3 | - |  |
| Weight | kg | 11.2 | 11.2 |  |
| Weight-for-age | SD | -2.3 | - |  |
| Weight-for-Height | SD | -1.2 | - |  |
| **Metabolic control** | |  |  |  |
| Glucose | mmol/L | 3.6 | - |  |
| Lactate | mmol/L | 1.0 | - |  |
| Uric acid | mmol/L | 0.12 | - |  |
| Triglyceride | mmol/L | 0.5 | - |  |
| Total cholesterol | mmol/L | 3.4 | - |  |
| Acetoacetate | mmol/L | - | - |  |
| Beta-OH-butyrate | mmol/L | - | - |  |
| **Cholestatic and liver parameters** | |  |  |  |
| AST | U/L | 40 | - | 33 |
| ALT | U/L | 26 | - | 17 |
| Ammonia | µmol/L | 40 | - |  |
| GGT | U/L | - | - | 7 |
| AP | U/L | - | - |  |
| Total bilirubin | µmol/L | 4 | - |  |
| Direct bilirubin | µmol/L | - | - |  |
| Thrombocytes | 10^9/L | - | - |  |
| APTT | sec | 40 | - |  |
| PT | sec | 12 | - |  |
| Albumin | g/L | - | - | 44 |
| Hepatomegaly** |  | No | - |  |
| Splenomegaly** |  | No | - |  |
| Portal hypertension |  | - | - |  |
| Ascites** |  | No | - |  |
| Cirrhosis |  | No** | - |  |
| **Muscle involvement** | |  |  |  |
| CK | U/L | 54 | - | 53 |
| **Cardiac involvement** | |  |  |  |
| NT-pro-BNP | ng/L | 57 | - |  |
| ECG |  | No | - |  |
| US |  | No | - |  |

**Patient 11**

Patient 11 presented clinically with arthrogryposis multiplex. She was managed with CNGDF after a severe hypoglycemia at the age of two years. By the age of 2.8 years, she received the genetic diagnosis of GSD IV by next generation panel sequencing. At the age of 3.3 years, she was prescribed a ketogenic diet in another metabolic centre. Simultaneously, she was affected by an adenovirus infection and the parents reported that subsequently liver functions worsened, and she developed ascites.

The parents of Patient 10 knew her parents and informed them about the diet in their daughter. She was referred to our liver GSD center of expertise for dietary evaluation and seen at the age of 5.1 years. At that time, ALT, AST and CK were 110, 196 and 47 U/l, respectively, and pre-albumin was low (0.10 g/l; reference 0.2 - 0.4 g/l). Liver functions of synthesis (albumin 36 g/L; APTT 41 sec; PT 16.1 sec) and detoxification (ammonia 53 µmol/l) were slightly abnormal, biochemically there was no cholestasis. Urinary excretion of tetraglucoside was 18.44 µmol/mmol kreat (normal < 4 µmol/mmol kreat). At abdominal ultrasound there was splenomegaly without ascites. Clinically and biochemically, there were no signs of cardiomyopathy.

Based on (1) profiles of CGM, (2) ketone measurements (max 1.4 mmol/l during the day; morning ketones were 0.6, 0.4 and 0.8 mmol/l), (3) weight-for-height (-2.2 SD) and (4) the low prealbumin concentrations, the speed of CNGDF was increased. This enhanced the nocturnal protein and carbohydrate intake to 1.7g/kg and 3.3 g/kg, respectively. Protein enrichment was carefully titrated due to elevated ammonia levels (53 µmol/L). Under these measures, the CGM profile improved and morning ketones decreased.

**Supplementary Table 11. Dietary Treatment and Outcomes for Patient 11.**

| **Prescribed diet** |  | **Diet history** | **Diet 1** |
| --- | --- | --- | --- |
| **Parameter** | **Unit** |  |  |
| Age | years | 5.1 | 5.1 |
| **Dietary treatment** | |  |  |
| Type of dietary treatment |  | CNGDF, PE | CNGDF, PE |
| Total energy | kcal/day | 784 | 870 |
| Total protein | g/day | 21.9 | 24 |
|  | g/kg/day | 1.5 | 1.7 |
|  | % of total energy | 11% | 11% |
| Dietary protein | g/day | 21.9 | 24 |
|  | g/kg/day | 1.5 | 1.7 |
| Protein from supplements | g/day | 0 | 0 |
|  | g/kg/day | 0.0 | 0.0 |
| Total fat | g/day | 36.3 | 40.3 |
|  | % of total energy | 42% | 42% |
| Total carbohydrates | g/day | 92.4 | 102 |
|  | % of total energy | 47% | 47% |
| Complex carbohydrates | g/day | 0 | 0 |
|  | g/kg/day | 0 | 0 |
| **Biometry** | |  |  |
| Height | cm | 104.0 | - |
| Height-for-age | SD | -1.9 | - |
| Weight | kg | 14.2 | 14.2 |
| Weight-for-age | SD | -2.7 | - |
| Weight-for-Height | SD | -2.2 | - |
| **Metabolic control** | |  |  |
| Glucose | mmol/L | 4.9 | - |
| Lactate | mmol/L | 1.7 | - |
| Uric acid | mmol/L | 0.15 | - |
| Triglyceride | mmol/L | 1.0 | - |
| Total cholesterol | mmol/L | 2.7 | - |
| Acetoacetate | mmol/L | - | - |
| Beta-OH-butyrate | mmol/L | - | - |
| **Cholestatic and liver parameters** | |  |  |
| AST | U/L | 196 | - |
| ALT | U/L | 110 | - |
| Ammonia | µmol/L | 53 | - |
| GGT | U/L | - | - |
| AP | U/L | 269 | - |
| Total bilirubin | µmol/L | 10 | - |
| Direct bilirubin | µmol/L | - | - |
| Thrombocytes | 10^9/L | - | - |
| APTT | sec | 41 | - |
| PT | sec | 16 | - |
| Albumin | g/L | 36 | - |
| Hepatomegaly** |  | No | - |
| Splenomegaly** |  | Yes | - |
| Portal hypertension |  | - | - |
| Ascites** |  | No | - |
| Cirrhosis |  | No** | - |
| **Muscle involvement** |  |  |  |
| CK | U/L | 47 | - |
| **Cardiac involvement** |  |  |  |
| NT-pro-BNP | ng/L | 55 | - |
| ECG |  | No | - |
| US |  | No | - |

**Patient 12**

Patients 12-14 were reported elsewhere (Szymanska 2018) but updated and available dietary data were added.

In brief summary, this is a case of a female patient with severe course of GSD type IV presenting with liver failure and neuro-muscular symptoms since neonatal period. At the age of 5 weeks laboratory diagnostics revealed transaminase elevation, cholestasis (bilirubin levels: total and direct 32.7 μmol/l and 14.3 μmol/l, respectively) and hyperlipidemia. When she was 9 months, failure to thrive (body weight -2.0 SD, height -3.5 SD), delayed psycho-motor development and hepatosplenomegaly were present. Additionally, portal hypertension with esophageal varices (bleeding 3 times and needed endoscopic intervention), muscle hypotonia of the lower limbs, and hypertrophic cardiomyopathy developed.

The decision of LT was made, and the surgery was performed when the girl was 22 months old. Histopathological examination of the liver explant revealed complete micronodular cirrhosis, sparse diffuse lymphocytic infiltration and presence of large granular PAS-positive intracytoplasmic inclusions located in numerous hepatocytes. On transmission electron microscopy of the liver aggregates with amylopectin deposition were found. Enzymatic tests showed brancher deficiency both in leukocytes and in the liver but not in the fibroblasts. Post-transplantation diagnosis of GSD IV was established therefore no dietary management was introduced.

Following LT, the patient’s hepatic function and nutritional status have improved, and although her mental development was good, the motor skills were still deeply delayed. Hypotonia and muscle atrophy disabled her – she did not walk at all. At the age of 6.5, the girl was admitted to the hospital due to sepsis and acute respiratory failure, and after 6 months at the intensive care unit she died.

**Patient 13**

A case of GSD IV patient with hepatic symptoms since infancy and moderate course of the disease, which has improved since dietary management was commenced. The diagnosis of GSD IV was established at the age of 26 months based on both enzymatic tests and histopathology. Liver core needle biopsy showed moderate fibrosis having no cirrhotic stage of nodular transformation. Intrahepatocyte inclusions had the same morphological and staining characteristics as in P12.

Once the diagnosis was established, dietary treatment based on high protein diet with restriction of non-utilizable sugars and regular food intake was introduced. The boy has been following check-up visits every 3 months at the beginning, and every 6 months afterwards.

The patient is 19 years old now and in good health. Neither liver complications nor growth and puberty disorders have been observed so far. He deals with life activities without any limitations.

**Supplementary Table 12. Dietary Treatment and Outcomes for Patient 13.**

| **Prescribed diet** |  | **Diet history** | **Diet 1** |
| --- | --- | --- | --- |
| **Parameter** | **Unit** |  |  |
| Age | years | 4 | 15 |
| **Dietary treatment** | | | |
| Type of dietary treatment |  |  | PE |
| Total energy | kcal/day | 870 | 2357-2737 |
| Total protein | g/day | 53 | 100-105 |
|  | g/kg/day | 1.0 | 2.0-2.5 |
|  | % of total energy | 10% | 12% |
| Dietary protein | g/day | 53 | 100-100.5 |
|  | g/kg/day | 1.0 | 2.0-2.5 |
| Protein from supplements | g/day | 0 | 20.0 |
|  | g/kg/day | 0.0 | 0.4 |
| Total fat | g/day | 37.0 | 40.0 |
|  | % of total energy | 42% | 42% |
| Total carbohydrates | g/day | 94 | 100 |
|  | % of total energy | 47% | 47% |
| Complex carbohydrates | g/day | 0 | 0 |
|  | g/kg/day | 0 | 0 |
| **Biometry** | | | |
| Height | cm | - | 176 |
| Height-for-age | SD | - | - |
| Weight | Kg | - | 66 |
| Weight-for-age | SD | - | - |
| Weight-for-Height | SD | - | - |
| **Metabolic control** | | | |
| Glucose | mmol/L | 4.9 | 4.2 |
| Lactate | mmol/L | - | - |
| Uric acid | mmol/L | - | - |
| Triglyceride | mmol/L | - | - |
| Total cholesterol | mmol/L | - | 4.3 |
| Acetoacetate | mmol/L | - | - |
| Beta-OH-butyrate | mmol/L | - | - |
| **Cholestatic and liver parameters** | | | |
| AST | U/L | 319 | 38 |
| ALT | U/L | 263 | 48 |
| Ammonia | µmol/L | - | - |
| GGT | U/L | - | 19 |
| AP | U/L | - | 2.82 |
| Total bilirubin | µmol/L | - | 8.0 |
| Direct bilirubin | µmol/L | - | - |
| Thrombocytes | 10^9/L | - | - |
| APTT | Sec | - | 31.9 |
| PT | Sec | - | 12.1 |
| Albumin | g/L | - | - |
| Hepatomegaly** |  | Yes | No |
| Splenomegaly** |  | No | No |
| Portal hypertension** |  | No | No |
| Ascites** |  | No | No |
| Cirrhosis |  | No | No** |
| **Muscle involvement** | | | |
| CK | U/L | - | 224 |
| **Cardiac involvement** | | | |
| NT-pro-BNP | ng/L | - | - |
| ECG |  | Yes | Yes |
| US |  | Yes | Yes |

**Patient 14**

This is a case of GSD IV in a young adult presenting only with cardiological symptoms; left ventricular dysfunction associated with mitral insufficiency and complicated with multiform ventricular arrhythmia.

He has never experienced any hepatological symptoms. His liver enzymes have always been within normal ranges. The diagnosis of GSD IV was established during workup for his cardiological symptoms by whole exome sequencing which revealed a heterozygous *ACTN2* NM_001103.3:c.2497G>A:(p. Ala833Thr) variant inherited from his mother classified as a variant of uncertain significance. The additional potentially pathogenic variants were found in the *GBE1* gene in trans position. Once the molecular diagnosis was established, he was referred to our hospital for consultation. We performed further enzymatic diagnostics that confirmed the genetic diagnosis.

We recommended a high protein diet with limitation of sugars. Since the patient is an adult, we do not have any follow-up of him and we do not know whether he complies with the dietary management at the age of 27 years now.

**Patient 15**

The patient is 5 years old now and the first child of these healthy, non-consanguineous parents of Polish origin. He was born after caesarian section due to the growth restriction and risk of asphyxia, birth weight was 1.8 kg at a gestational age of 35 weeks. Labor was complicated with respiratory failure and Apgar score were 8/7/7 points. After birth arthrogryposis was diagnosed. Although his motor functioning was impaired, mental development was normal. Postnatal abdominal ultrasound did not reveal any abnormalities (both liver and spleen were not enlarged). Preliminary genetic diagnostics included karyotype which was normal 46XY and spinal muscular atrophy (SMA) was excluded. Since birth the child has had rehabilitation and he was equipped with ankle-foot orthoses.

At the age of 20 months due to pharyngitis, laboratory diagnostics was made and revealed elevated serum activity of liver enzymes (ALT 303 U/L, AST 501 U/L). On abdominal ultrasounds enlarged spleen without any other abnormalities was described. Viral causes (HCV, HBV, CMV, HIV) of hepatitis were excluded. Due to persistent increased serum aminotransferases concentrations and additional appearance of hepatomegaly, gastrological and metabolic diagnostics were performed. Laboratory tests showed increased GGT activity and elevated bile acids level, but CK, alfa-1-antitrypsin, serum levels of copper and iron, immunoglobulins and lactate level were within normal range values. Autoimmune hepatitis, celiac disease and tubulopathies were also excluded. Both ophthalmological examination and echocardiography did not reveal any abnormalities. In organic acids profile with GC-MS, increased levels of lactate, 3-hydroxyisovaleric acid, and fumaric acid were detected. Isoforms of transferrin and biotinidase activity were normal. Both acylcarnitines profile with tandem mass spectrometry and aminoacid profile did not reveal any pathological findings. Lysosomal storage diseases were excluded enzymatically. Fibroscan showed moderate fibrosis and mild steatosis.

At the age of 2.5 years, genetic testing using whole exome sequencing method was performed and in trans mutations in *GBE1* gene were detected, which confirmed the diagnosis of GSD IV. Once the diagnosis was made a protein enriched diet was introduced. Currently the boy is clinically stable, however, he developed portal hypertension for which regular endoscopic check-up visits are needed.

**Supplementary Table 13. Dietary Treatment and Outcomes for Patient 15.**

| **Prescribed diet** |  | **Diet history** | **Diet 1** |
| --- | --- | --- | --- |
| **Parameter** | **Unit** |  |  |
| Age | years | 2.5 | 2.5 |
| **Dietary treatment** | | | |
| Type of dietary treatment |  | None | PE |
| Total energy | kcal/day | - | 1000 |
| Total protein | g/day | - | 23.4 |
|  | g/kg/day | - | 1.8 |
|  | % of total energy | - | 12% |
| Dietary protein | g/day | - | 23.4 |
|  | g/kg/day | - | 1.8 |
| Protein from supplements | g/day | - | 0 |
|  | g/kg/day | - | 0.0 |
| Total fat | g/day | - | 39.0 |
|  | % of total energy | - | 40% |
| Total carbohydrates | g/day | - | 100 |
|  | % of total energy | - | 46% |
| Complex carbohydrates | g/day | - | 0 |
|  | g/kg/day | - | 0 |
| **Biometry** | | | |
| Height | cm | - | 91 |
| Height-for-age | SD | -2.0 | - 1.5 |
| Weight | kg | - | 13.0 |
| Weight-for-age | SD | -2.5 | - |
| Weight-for-Height | SD | - | - |
| **Metabolic control** | | | |
| Glucose | mmol/L | - | 5.8 |
| Lactate | mmol/L | - | - |
| Uric acid | mmol/L | - | 0.18 |
| Triglyceride | mmol/L | - | 0.67 |
| Total cholesterol | mmol/L | - | 3.5 |
| Acetoacetate | mmol/L | - | - |
| Beta-OH-butyrate | mmol/L | - | - |
| **Cholestatic and liver parameters** | | | |
| AST | U/L | 73 | 96 |
| ALT | U/L | 79 | 113 |
| Ammonia | µmol/L | - | - |
| GGT | U/L | - | 44 |
| AP | U/L | 200 | - |
| Total bilirubin | µmol/L | 7.2 | 9.7 |
| Direct bilirubin | µmol/L | - | 1,2 |
| Thrombocytes | 10^9/L | - | - |
| APTT | sec | 39.06 | 38.4 |
| PT | sec | 13.04 | 12.82 |
| Albumin | g/L | 46.6 | 46.9 |
| Hepatomegaly** |  | Yes | No |
| Splenomegaly** |  | Yes | Yes |
| Portal hypertension |  | No | Yes |
| Ascites** |  | No | - |
| Cirrhosis |  | No** | - |
| **Muscle involvement** |  |  |  |
| CK | U/L | - | - |
| **Cardiac involvement** | | | |
| NT-pro-BNP | ng/L | - | - |
| ECG |  | Yes | Yes |
| US |  | Yes | Yes |
